# Decoding and engineering temperature-sensitive lethality in *Ceratitis capitata* for pest control

**DOI:** 10.1073/pnas.2503604122

**Published:** 2025-07-07

**Authors:** Roswitha A. Aumann, Georgia Gouvi, Maria-Eleni Gregoriou, Tanja Rehling, Germano Sollazzo, Kostas Bourtzis, Marc F. Schetelig

**Affiliations:** ^a^Department of Insect Biotechnology in Plant Protection, Institute for Insect Biotechnology, Justus-Liebig-University Gießen, Gießen 35394, Germany; ^b^Liebig Centre for Agroecology and Climate Impact Research, International Atomic Energy Agency Collaborating Centre, Justus-Liebig-University Gießen, Gießen 35394, Germany; ^c^Insect Pest Control Section, Joint Food and Agriculture Organization of the United Nations/International Atomic Energy Agency Centre of Nuclear Techniques in Food and Agriculture, Department of Nuclear Sciences and Applications, International Atomic Energy Agency, Vienna 1400, Austria; ^d^Department of Sustainable Agriculture, Laboratory of Systems Microbiology and Applied Genomics, University of Patras, Agrinio 30100, Greece

**Keywords:** genetic sexing strain, mini-gene rescue, tsl, *lysine--tRNA ligase* (*Lysyl-tRNA synthetase*, *LysRS*), CRISPR/Cas gene editing

## Abstract

The Sterile Insect Technique (SIT) is a species-specific and environmentally friendly method for effectively controlling pest insect populations based on releasing reared, sterile insects into infested areas. Sex sorting in rearing facilities, enabling male-only releases, is necessary to ensure SIT programs are efficient, cost-effective and, in case of mosquito control, also safe. This can be greatly facilitated by genetic sexing strains (GSS), exhibiting sex-specific phenotypic markers. However, the development of GSS remains challenging. The construction of a *temperature-sensitive lethal* (*tsl*)-based GSS in the Mediterranean fruit fly (*Ceratitis capitata*) over three decades ago was considered a major breakthrough for SIT programs but was never successfully replicated in other pests. After over 30 y of research, we have pinpointed a specific mutation in the *C. capitata lysine--tRNA ligase* (*Lysyl-tRNA synthetase*, *LysRS*) gene responsible for the tsl phenotype. Introducing this specific mutation into a wild-type strain produced full embryonic lethality under heat stress, replicating the original mutant phenotype. The random integration of a *LysRS* minigene reversed this effect. The high conservation of *LysRS* among insects suggests that tsl-based GSS could be expanded to multiple pest species and extend applications of SIT programs for disease prevention and the protection of agriculture.

## Significance

This study identifies the long-sought gene responsible for the temperature-sensitive lethal (tsl) phenotype in *Ceratitis capitata* genetic sexing strains (GSS). GSS are crucial for making Sterile Insect Technique programs efficient and cost-effective, but their development is challenging when relying solely on classical genetics. By identifying the *lysine--tRNA ligase* (*Lysyl-tRNA synthetase*, *LysRS*) gene as the molecular basis of the tsl trait, we replicated the tsl phenotype through precise genetic engineering and demonstrated its reversibility with a *LysRS* minigene. Our research provides a blueprint for engineering a conditional marker and its rescue, which can be utilized to create GSS in insect pests of medical and agricultural importance in a time-efficient manner using modern gene editing techniques.

The development of the first *temperature-sensitive lethal* (*tsl*)-based genetic sexing strain (GSS) for the Mediterranean fruit fly (medfly, *Ceratitis capitata*), a major agricultural pest, revolutionized the Sterile Insect Technique (SIT) (1). SIT is an environmentally friendly and species-specific insect pest control method that has been applied successfully in various species, with the largest programs targeting medfly (2, 3). It involves breeding and releasing sterilized male insects to compete with wild males in the field, thus reducing the pest population (4). Releasing only male insects is essential for efficiency and cost-effectiveness and can be achieved through GSS, which allow male–female separation (“sexing”) based on specific traits (1, 5). GSS are typically engineered by combining recessive morphological markers with a sex chromosome/autosome translocation (1, 6). Natural pupal color mutations have been utilized to develop GSS in various fruit fly species (7–11), and (functional) genomics, transcriptomics, cytogenetics, and bioinformatic analyses have facilitated the identification of the genetic basis of *white pupae* (*wp*) and *black pupae* in several tephritid fruit flies (12–14). Typically, females are homozygous for the mutation, displaying the mutant phenotype, while males, carrying a translocation that links the wild-type (WT) allele to the Y chromosome, develop WT puparia. However, sexing in the pupal stage is costly, has an approximately 5% error rate, and can impair adult flight ability (15). Adding a pandevelopmental tsl trait is unique to the medfly GSS VIENNA-7 and VIENNA-8. In these strains, white puparia females (*wp^–^*/*tsl^–^*) die at elevated temperatures (34 to 35 °C) at any developmental stage, while brown puparia males survive under these conditions (1). The *tsl*^–^-based embryo sexing through water bath immersion is more cost-effective and accurate than mechanical pupal sorting. Furthermore, the *tsl*-based approach is particularly noteworthy for preserving male fitness in mass-rearing systems (1, 16–18). Due to its proximity and being tightly linked to the tsl trait, the *wp*^–^ mutation serves as a check for proper heat treatment and helps to identify recombination events in these GSS, essential for maintaining colony integrity in rearing facilities (1, 16, 17, 19). The medfly tsl trait was discovered during a mutagenesis screen more than 30 y ago (20, 21). It is known that the causative mutation is located on the right arm of chromosome 5, downstream of *wp,* outside the pericentric D53 inversion, in proximity to the (molecularly unidentified) *Sergeant-2* (*Sr^2^*) gene, and upstream of the *glucose-6-phosphate 1-dehydrogenase* (*Zw*) gene (1, 19, 22, 23). The mutation is recessive and exhibits a maternal effect, as it has been demonstrated that F_1_ embryos from crosses between homozygous *tsl* mutant females and WT males show sensitivity to elevated temperatures during the early stages of development (i.e., when the embryo is still dependent on the egg cytoplasm and has not yet activated its own zygotic genetic material), whereas embryos from reciprocal crosses do not exhibit this sensitivity (1, 18, 21). It results in reduced egg hatching rates after 24 h at 31 °C, and full embryonic lethality after 24 h at 34 °C (1, 24). However, its molecular basis, including the number of genes involved, remains unknown (1). The recently proposed “generic approach” to create “neo-classical GSS” by transferring known, suitable markers to other insect pests through gene editing could greatly expand SIT applications (23, 25, 26). Therefore, identifying the tsl trait in medfly, recognized as superior to other known GSS markers, is of significant interest. A recent study investigated the so-called *tsl* genomic region, resulting in the identification of several tsl candidate genes, including *deep orange* (*dor*) (23). Although targeted mutagenesis of *dor* resulted in tsl phenotypes, they did not precisely mirror those of the original *tsl^–^* strain (27), prompting us to continue and revise the candidate screening.

Here, we conclusively identify the highly conserved *lysine--tRNA ligase* (*Lysyl-tRNA synthetase*, *LysRS*; also known as *Syk* or *spleen tyrosine kinase*) gene as the cause of the medfly tsl phenotype, solving a more than 30-y-old mystery. *LysRS* is located within the designated *tsl* genomic region and features a point mutation in its core domain in strains with the “original” tsl mutant phenotype. We recreated *LysRS* mutants by CRISPR/Cas9 homology-dependent repair (HDR) and observed an equivalent recessive phenotype (reduction in egg viability after 24 h at 31 °C, full embryonic lethality at 34 °C, and a prominent maternal effect). A complementation assay with the original *tsl^–^* mutant strain confirmed that *LysRS* is the causative gene. We also showed that the phenotype can be restored by randomly integrating an engineered *LysRS* minigene, paving the way to develop *tsl*-based sexing strategies in other pest insects to expand the applications of SIT programs.

## Results

### Selection of *LysRS* as *tsl* Candidate Gene.

The identification of *LysRS* as a candidate gene was achieved through a comprehensive screening of genes that feature nonsynonymous mutation(s), are located within the previously described *tsl* genomic region (23), and have similar properties to known medfly tsl characteristics (for detailed information, see *SI Appendix*, Supporting Text S1 and Fig. S1). The *LysRS* gene (NCBI ID LOC101451416), carrying a single-nucleotide polymorphism (SNP) that replaces histidine with tyrosine (H516Y in XP_004536422.1 and H464Y in XP_004536423.1; *SI Appendix*, Fig. S2), was selected as the most promising candidate because i) it is predicted to be positioned in the previously delineated *tsl* region (1, 19, 22, 23); ii) it is highly conserved among insects (*SI Appendix*, Fig. S3), with orthologs present in all 255 insect species aggregated in OrthoDB, and has essential and conserved functions explaining the lethality of homozygous mutations (28, 29); iii) the mutation is located within the highly conserved LysRS core domain (PLN025502 domain; *SI Appendix*, Fig. S2), suggesting an important function; iv) the substitution was predicted to affect the native protein function with a PredictProtein score of +26 (score ranges from –100 to +100, +26 represents strong neutral to strong impact); and v) the orthologous gene in *D. melanogaster* (CG12141) is ubiquitously and continuously expressed, matching the pandevelopmental nature of the tsl trait, and shows high RNA expression level in ovaries and early embryonic stages, suggesting maternal deposition. Sequencing the *LysRS* gene in homozygous *tsl^–^* mutants (*wp^–^*/*tsl^–^*, D53, VIENNA-7, and VIENNA-8 females), heterozygous *tsl^–^* mutants (VIENNA-7, VIENNA-8, and VIENNA-8 *Sr^2^* males), and WT flies of strains Egypt II (EgII) and Benakeion confirmed that the candidate matches the tsl phenotypes and *tsl* genotypes of these strains (temperature sensitive or resistant and mutation homozygous, heterozygous, or WT, as appropriate).

### Analysis of Medfly *LysRS*.

**Fig. 1.**
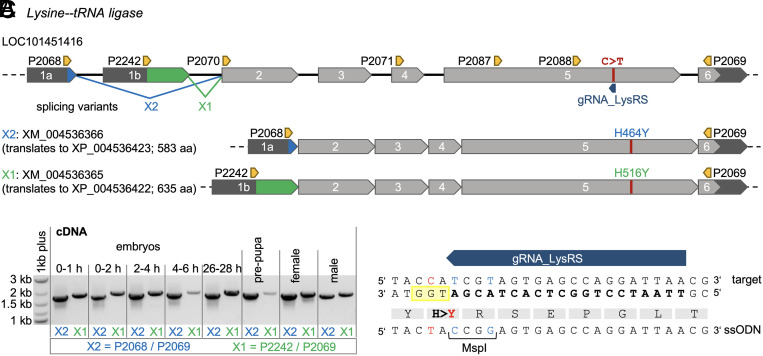
*Lysine--tRNA ligase* (*Lysyl-tRNA synthase*, *LysRS*) gene structure, alternative splicing variants, and experimental strategy to create the *LysRS_H>Y* mutation. (*A*) *LysRS* (LOC101451416) gene structure and alternative splicing transcripts. UTRs are depicted in dark gray, common CDS exons in light gray, and CDS exons specific to a transcript variant in blue (X2, corresponding to XM_004536366.3, which translates into protein isoform XP_004536423.1) and green (X1, corresponding to XM_004536365.3, which translates into protein isoform XP_004536422.1), respectively. The exon count, the position of the target mutation (red), and the positions of the single guide RNA (gRNA_LysRS; dark blue) and primers (orange) are indicated. Not to scale. (*B*) Verification of alternative splicing variants in cDNA samples from different developmental stages of WT EgII. An uncropped version of the gel is provided in *SI Appendix*, Fig. S5. (*C*) Experimental strategy to create the *LysRS_H>Y* mutation in the WT EgII strain, showing the gRNA target sequence (bold letters) and orientation, the position of the protospacer adjacent motif (PAM, yellow box), the amino acid sequence, and part of the repair template (ssODN_LysRS_H>Y) including the MspI site. Desired mutations distinguishing between the genomic target site and the ssODN are highlighted in red (functional mutation) or blue (silent mutation).

In situ hybridization and cytogenetic analysis showed that *LysRS* is located on the right arm of *C. capitata* chromosome 5, in region 77A/B of the salivary gland polytene chromosome map (*SI Appendix*, Fig. S4), which matches the previously predicted location of the *tsl* gene (1, 19, 22, 23). We amplified and sequenced *LysRS* cDNA from early and late embryonic, prepupal, and adult stage samples and confirmed the NCBI-annotated exon/intron structure and sequence, as well as the predicted cytoplasmic and mitochondrial isoforms produced by alternative splicing (30, 31), in all tested samples (Fig. 1 *A* and *B*). The presence of *LysRS* transcripts in embryos aged 0 to 1 h [i.e., before the onset of zygotic expression (32)] suggests maternal deposition in *C. capitata* (Fig. 1*B*).

### Generation of *LysRS* Mutants.

To determine whether the H > Y substitution in the LysRS core domain is (solely) responsible for the tsl phenotype in *wp^–^*/*tsl^–^* mutants, the candidate mutation, along with two silent mutations, was introduced into the *LysRS* gene of the temperature-resistant WT strain EgII (24) by CRISPR/Cas9-HDR (Fig. 1*C* and *SI Appendix*, Supporting Text S2). We applied three different crossing strategies in parallel to establish homozygous *LysRS_H>Y ^CRISPR^* mutant strains (*SI Appendix*, Fig. S6 and Supporting Text S2).

In the first strategy, we introduced the *LysRS_H>Y^ CRISPR^* mutation into the chromosome 5 balancer strain 68B, which carries the balancer chromosome FiM1 (33). FiM1 has three overlapping pericentric inversions that suppress recombination along the length of the fifth chromosome and is marked with, among others, the homozygous lethal *Sergeant-2* (*Sr^2^*) mutation. The presence of the *Sr^2^* mutation is visually indicated by three, instead of two, white stripes on the abdomen (33). This strategy ensured the maintenance of the *LysRS_H>Y^CRISPR^* in a heterozygous form, allowing for the preservation of the putative lethal mutation. The retrieved homozygous mutants in the genomic background of the balancer strain (without the balancer chromosome itself) could be successfully maintained and are hereafter described as *LysRS_H>Y^ CRISPR^[B]*. In the second strategy, we established a homozygous line carrying only the paternal genomic background (EgII), hereafter described as *LysRS_H>Y^ CRISPR^[E]*. The fertility (hatching), fecundity (number of eggs), and developmental survival rates of this strain are equivalent to the WT EgII strain (*SI Appendix*, Figs. S7–S9). To explore the effect of a different genetic background on the strains’ performance, we implemented a third crossing strategy: *LysRS_H>Y ^CRISPR^[E]* mutants were crossed to the original homozygous *wp^–^*/*tsl^–^*strain to induce recombination between the *wp^–^* and *LysRS* loci. Recombinants were inbred to establish a double homozygous strain (*wp^–/–^ LysRS_H>Y^ CRISPR/CRISPR^*; *SI Appendix*, Fig. S6*B*), hereafter described as *LysRS_H>Y^ CRISPR^[t]*.

Notably, homozygous *LysRS_H>Y^CRISPR^[B]* and *LysRS_H>Y^CRISPR^[E]* mutants could be reared continuously and successfully at 23 °C, but initially exhibited reduced fertility and fecundity at 25 °C (*SI Appendix*, Fig. S6). Following various generations of backcrossing and sustained rearing at 25 °C, accompanied by fluctuations in hatching rates, survival rates eventually stabilized and became comparable to WT levels (*SI Appendix*, Fig. S9). In contrast, *LysRS_H>Y^CRISPR^[t]* mutants could be successfully reared at 25 °C after a single initial decline in survival rates (*SI Appendix*, Fig. S6 and Supporting Text S2).

### Temperature Sensitivity Characteristics.

**Fig. 2.**
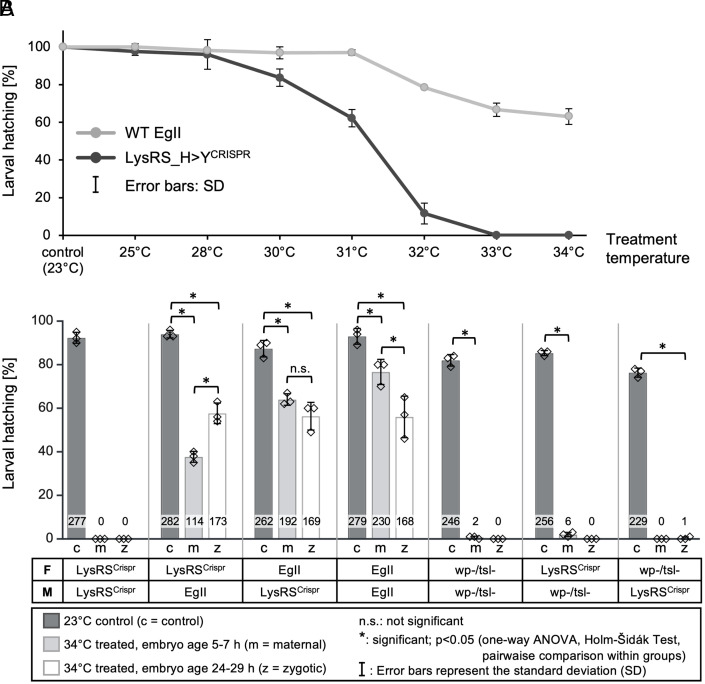
Larval hatching rates of mutant and WT strains after different heat treatments. (*A*) Larval hatching rates (shown as mean ± SD) of *LysRS_H>Y^CRISPR^[E]* and WT EgII after 24-h treatments at different temperatures, relative to the control at 23 °C. The mutants show significantly greater mortality at 31 °C (one-way ANOVA, *P* < 0.05. Error bars represent the SD of the measurements), and full lethality starting at 34 °C. The 24-h heat treatments at 25, 28, 30, 31, 32, 33, and 34 °C were applied to 300 embryos aged 24 to 29 h (*n* = 3 × 100 eggs per strain) of the homozygous *LysRS_H>Y^CRISPR^[E]* and WT EgII strains. An experiment using homozygous *LysRS_H>Y^CRISPR^[B]* mutants yielded similar results (*SI Appendix*, Fig. S10). (*B*) Larval hatching rates of reciprocal crosses of *LysRS_H>Y^ CRISPR^[E]*, WT EgII, and *wp^–^*/*tsl^–^* mutant flies after heat treatment at 34 °C, shown as mean ± SD. LysRS^CRISPR^ mutants (= *LysRS_H>Y^ CRISPR^[E]*) were crossed to WT EgII or *wp^–^*/*tsl^–^* flies. The crosses (F = female parent, M = male parent) and strains are shown below the graph. For each biological replicate, we collected 3 × 100 F_1_ embryos (technical replicates) for 5 h, and either kept them at 23 °C at all times (c = control), kept them at 23 °C for 2 h and then switched them to 34 °C for 24 h (embryonic age 5 to 7 h, m = maternal treatment), or kept them at 23 °C for 24 h and switched them to 34 °C for 24 h (embryonic age 24 to 29 h, z = zygotic treatment). The larval hatching rate is shown as a percentage along with the absolute number of larvae (inside the bar). Individual datapoints of each bar are indicated (diamonds). One-way ANOVA was used to determine whether differences within groups were statistically significant (*P* < 0.05, Holm-Šidák test) or not (n.s. = not significant). Error bars represent the SD of the measurements. The experiment was carried out three times (biological replicates) with similar results. An experiment with homozygous *LysRS_H>Y^CRISPR^[B]* mutants yielded similar results (*SI Appendix*, Fig. S11).

Incubating homozygous CRISPR mutant and WT EgII embryos (aged 24 to 29 h) at temperatures ranging from 25 to 34 °C for 24 h revealed a gradual decline in hatching rates for WT EgII embryos. In contrast, *LysRS_H>Y ^CRISPR^* mutant embryos exhibited a significant drop in survival starting at 31 °C and complete lethality at 34 °C (Fig. 2*A* and *SI Appendix*, Fig. S10), confirming that the *LysRS_H>Y^CRISPR^* mutation induces a tsl phenotype. Reciprocal crosses between the *LysRS_H>Y ^CRISPR^* mutant and the WT EgII or *wp^–^*/*tsl^–^* mutant strains were set up to evaluate the genetic properties of the mutation, and resulting embryos were incubated for 24 h at 34 °C starting at the early or late stage of embryogenesis (aged 5 to 7 h and 24 to 29 h, respectively; Fig. 2*B* and *SI Appendix*, Fig. S11). The *LysRS_H>Y ^CRISPR^* mutants were fully susceptible to both heat treatments, whereas the heterozygous offspring of the WT EgII × *LysRS_H>Y ^CRISPR^* crosses survived the treatments, indicating a recessive phenotype. Furthermore, offspring of homozygous *LysRS_H>Y ^CRISPR^* females crossed to WT males showed significantly lower survival rates following early embryonic treatment than late treatment. In contrast, offspring of the reciprocal cross (mutant males crossed to WT females) showed comparable survival rates after both treatments, suggesting a maternal effect. Offspring of the *wp^–^*/*tsl^–^*× *LysRS_H>Y ^CRISPR^* crosses showed no larval hatching or very low rates after the heat treatments, a tsl phenotype comparable to the original homozygous *wp^–^*/*tsl^–^*mutant. This indicates that the tsl phenotype in both strains is caused by mutations in the same gene.

### Rescue with a *LysRS* Minigene.

**Fig. 3.**
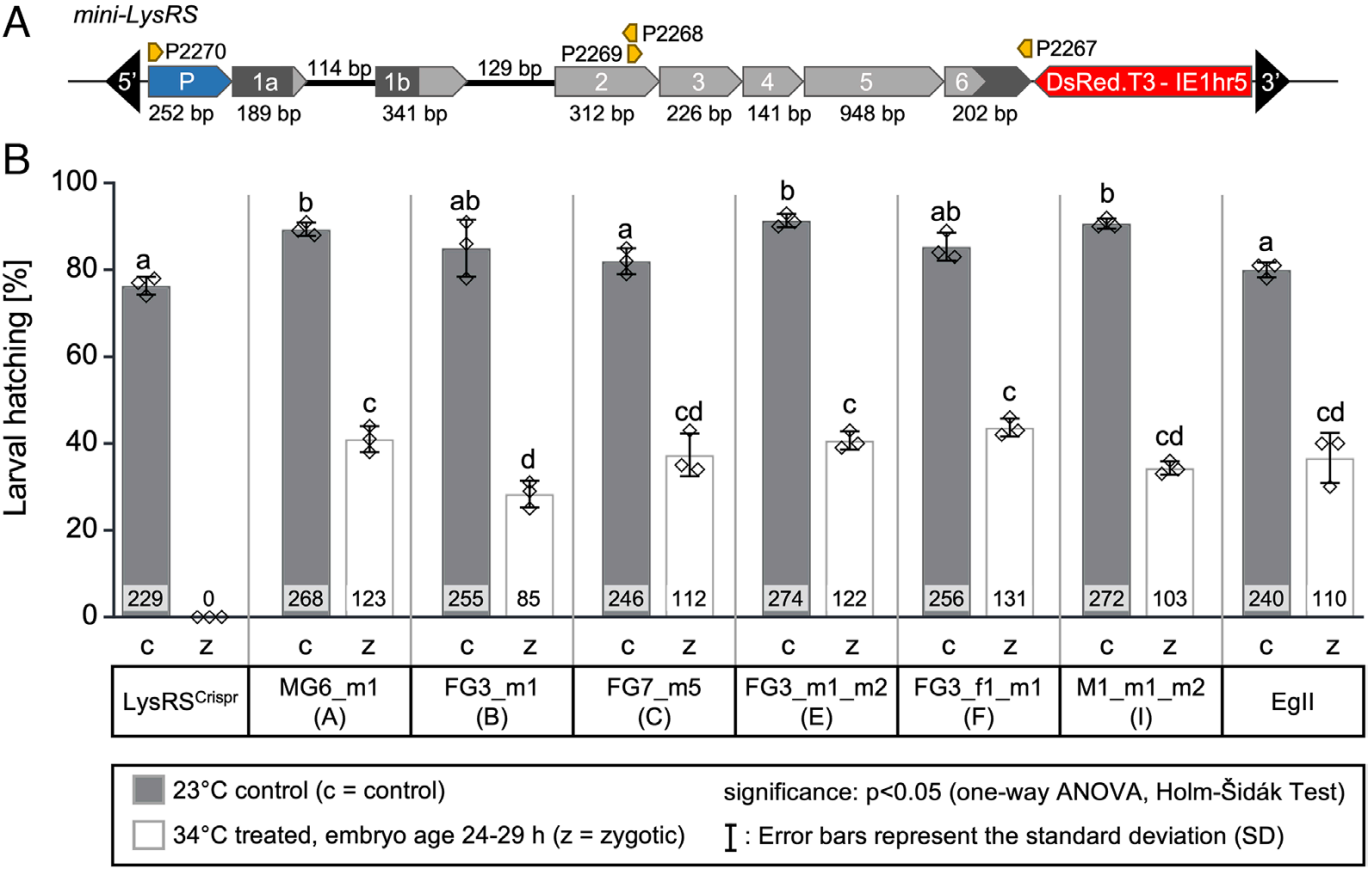
*mini-LysRS rescue* strains: minigene construct and larval hatching rates after heat treatment. (*A*) Map of the *LysRS* minigene in the *M6643 piggyBac* transformation vector, used to generate *mini-LysRS rescue* mutant strains (*piggyBac* ends, black arrows; UTRs, dark gray; CDS, light gray; *IE1hr5-DsRed.T3-SV40*, red; exon count), including the alternative splicing variants (exon 1a/1b with introns), the putative promoter region (blue), the size of the genetic elements (bp = base pairs), and the position of the primers (orange) used for amplification and cloning. Not to scale. The putative promoter region is shown in more detail in *SI Appendix*, Fig. S12. (*B*) Larval hatching rates of homozygous *mini-LysRS rescue* mutant strains after heat treatment, shown as mean ± SD. We collected 3 × 100 embryos over a period of 5 h and either kept them at 23 °C at all times (c = control), or kept them at 23 °C for 24 h before switching to 34 °C for 24 h (embryonic age 24 to 29 h, z = zygotic treatment). The larval hatching rate is shown as a percentage along with the absolute number of larvae (inside the bar). Individual datapoints of each bar are indicated (diamonds). One-way ANOVA was used to determine whether differences within groups were significant (*P* < 0.05, Holm-Šidák test; significant differences are indicated by different letters) or not (identical letters). Error bars represent the SD of the measurements. The experiment was carried out twice (biological replicates) with similar results (*SI Appendix*, Fig. S14).

Next, we engineered a minigene (*mini-LysRS*) to assess its ability to rescue the tsl phenotype. The *mini-LysRS* (2,854 bp) was assembled from the putative promoter region, the annotated 5′ UTR, exons 1a, 1b, and 2 with their respective introns, the remaining coding sequence without introns, and the 3′ UTR (*SI Appendix*, Fig. S12 and Supporting Text S3). Together with a DsRed marker (2,033 bp), this sequence was inserted into a *piggyBac* transformation vector (Fig. 3*A*) for transgenesis of *LysRS_H>Y^CRISPR^[E]* embryos (*SI Appendix*, Supporting Text S3).

Six families could be maintained [MG6_m1 (A), FG3_m1 (B), FG7_m5 (C), FG3_m2_m1 (E), FG3_f1_m1 (F) and M1_m1_m2 (I); *SI Appendix*, Supporting Text S3], were verified as single-copy strains by droplet digital PCR (ddPCR) in generation G_3_, and were inbred to homozygosity (*SI Appendix*, Fig. S13). We were able to resolve the genomic position of the construct by inverse PCR (iPCR) in four of six families, revealing independent autosomal integration sites for MG6_m1 (A), FG7_m5 (C), FG3_f1_m1 (F), and M1_m1_m2 (I) on scaffolds 30, 18, 325, and 511, respectively (GCA_000347755.4). Integration sites could not be resolved for FG3_m1 (B) or FG3_m2_m1 (E). Temperature sensitivity tests with embryos aged 24 to 29 h (zygotic genome active) representing all homozygous *mini-LysRS* mutant strains were conducted to test their temperature sensitivity. The tsl phenotype was rescued in all six tested strains, with larval hatching rates comparable to the WT control (Fig. 3*B* and *SI Appendix*, Fig. S14).

## Discussion

The tsl phenotype is a unique success factor of the medfly GSS currently used in mass-rearing facilities worldwide. It enables the selective killing of female embryos using a simple heat treatment, which significantly reduces costs and effort compared to GSS based on pupal markers (1). Extending this advantageous phenotype to other pest insects could greatly expand SIT applications. However, it is necessary to identify the causal *tsl* gene(s) to allow the construction of *tsl*-based GSS in other species using a “generic approach” (25, 26). Therefore, efforts continue to understand and apply tsl traits across species (23, 27, 34–37).

In this study, we identified a single amino acid substitution at a highly conserved position in the C-terminal catalytic domain of the medfly *LysRS* gene in *tsl^–^* mutant strains. LysRS is a class II tRNA ligase (subgroup IIb) that participates in various multienzyme complexes, has cytoplasmic and mitochondrial isoforms, and is a necessary component of several biological processes (28, 29, 31). We functionally characterized the candidate mutation by regenerating the precise histidine-to-tyrosine substitution in a WT strain, confirming that these de novo mutants have characteristics identical to the original tsl mutant phenotype. Three major lines of evidence support the hypothesis that *LysRS* is the elusive *C. capitata tsl* gene. First, it is located in the *tsl* genomic region, precisely at the predicted position. Second, the *LysRS_H>Y^CRISPR^* phenotype exactly mirrors that of the original *tsl^–^* strain regarding temperature sensitivity and lethality, recessive inheritance, and maternal effect. Third, crossing *LysRS_H>Y^CRISPR^* and *tsl^–^* flies produced fully susceptible temperature-sensitive embryos, indicating that the mutations are identical rather than complementary.

During laboratory-scale rearing, the survival, fertility, and fecundity rates of the *LysRS_H>Y^CRISPR^[E]* mutant were comparable to those of the WT EgII strain, fulfilling an essential criterion for mass-rearing (*SI Appendix*, Figs. S7–S9). Interestingly, *LysRS_H>Y^CRISPR^[E]* mutants adapted to a 25 °C rearing temperature only after several generations of backcrossing and sustained exposure to this condition, whereas *LysRS_H>Y^CRISPR^[t]* mutants adapted more rapidly. Fitness and lethality tests from various *tsl^–^* mutant strains and *tsl*-based GSS indicate that the tsl phenotype and the strain performance show a certain degree of variability and can change over time (16, 24). Factors such as introgression into different genetic backgrounds, spontaneous mutations, genetic bottlenecks, and bacterial endosymbionts can significantly alter the characteristics and phenotypes of a strain (16, 24, 38–40). As the original tsl phenotype was discovered during an ethyl methanesulfonate (EMS) mutagenesis screen, a method inducing numerous random mutations across the genome (20, 21), it is possible that an additional mutation, generated alongside the *LysRS* mutation during the EMS treatment, contributes to the tolerance of the *wp^–^*/*tsl^–^*strain to 25 °C rearing conditions. *LysRS_H>Y^CRISPR^[t]* mutants may have incorporated this additional mutation, potentially explaining their rapid adaption. In contrast, for *LysRS_H>Y^ CRISPR^[E]* mutants, sustained breeding at 25 °C may have been necessary to allow for the selection of spontaneous beneficial compensatory mutations, accounting for their protracted adaption. Notably, both *LysRS_H>Y^ CRISPR^[E]* and *LysRS_H>Y^ CRISPR^[t]* mutants retained their prominent tsl phenotype (reduced survival rates after 24 h at 31, 32, and 33 °C, and complete embryonic lethality after 24 h at 34 °C, 35 °C, and 36 °C), even after adapting to 25 °C rearing conditions. Before considering the *LysRS_H>Y^ CRISPR^[E]* strain for SIT applications, comprehensive biological quality assessments under (semi-) mass-rearing conditions will be necessary to ensure robustness and long-term efficiency.

Based on its striking tsl phenotype and good rearing performance, the mutation in the *LysRS* gene may be suitable for the development of more efficient and cost-effective GSS in other pest species by introducing the same amino acid substitution in *LysRS* orthologs using CRISPR/Cas9 HDR-mediated mutagenesis (“neoclassical GSS”). The comparison of LysRS across different SIT target species in silico has shown that LysRS is not only present in all species tested thus far, but its protein sequence is highly conserved (*SI Appendix*, Fig. S3). Notably, the histidine at the target position is universal in all 500 species that have been examined (NCBI blastp search vs insects, taxid 50557). This high level of conservation suggests that the same mutation may induce similar tsl phenotypes in various pest species, thus enabling the development of GSS using a generic approach (25, 26). Like classical GSS, “CRISPR-engineered GSS” or “neoclassical GSS” would require not only a suitable marker gene but also a male-specific rescue, e.g., by integrating a minimal WT allele gene fragment at an appropriate position on the male-specific chromosomes by HDR-mediated knock-in, preferably at a site near the maleness factor (41, 42). Toward this goal, we already created a *mini-LysRS* rescue construct and inserted it at six different autosomal genomic positions. Double homozygous mutants carrying this rescue construct and the *LysRS_H>Y^CRISPR^* mutation showed resistance to elevated temperature (34 °C), indicating that the construct is fully functional. The identification of suitable target sites that ensure robust expression and stable integration of the construct on the highly repetitive and heterochromatic *C. capitata* Y chromosome (43) will be the next challenge.

It is to be determined whether analogous mutations in *LysRS* orthologs of other pest species will trigger tsl phenotypes comparable to that observed in the medfly, and if a generic approach based on *LysRS* will be feasible. Previous attempts to engineer strains using temperature-sensitive mutations derived from the *D. melanogaster* genes, such as *transformer-2*, *deep orange,* or *shibire*, have revealed that phenotypic manifestation and permissive/restrictive temperatures of ts mutations may vary among species (27, 34, 35, 44). So far, temperature-sensitive strains used to develop GSS, such as in *C. capitata* (1, 21), or those with potential for future use [e.g., *Anopheles arabiensis* (37)], have been generated through EMS mutagenesis—a time-consuming, nontargeted approach. In contrast, establishing the *LysRS_H>Y^CRISPR^* mutant strain by introducing a specific single-point mutation required only one embryonic microinjection attempt and three generations of rearing and genotyping. Compared to the only other engineered temperature-sensitive embryonic-lethal mutant strain in medfly, *dor_51dup* [which is based on a random mutation in the *deep orange* gene and would also serve as a suitable marker for a neoclassical GSS (27)], the *LysRS_H>Y^CRISPR^* strain stands out due to its relatively modest restrictive temperature of 34 °C (compared to 36 °C for *dor_51dup*), which allows for high survival rates of heterozygous mutants (e.g., GSS males). Furthermore, creating a *tsl* allele in *LysRS* required only a single nucleotide change, unlike the 51 bp duplication coupled with a specific SNP mutation needed in *dor*. Notably, our approach also avoids transgenic elements and relies solely on endogenous genes, likely minimizing regulatory complexity. Despite ongoing revisions to genetically modified organism (GMO) regulations in many countries, it appears that most classifications will still focus on the presence of foreign DNA in the final product. Edits generated through genome-editing techniques, on the other hand, are typically categorized into three site-directed nuclease (SDN) classes (45, 46). Consequently, transgenic sexing strains, developed for several pest insect species already over a decade ago (47–52), likely remain regulated as GMOs in most countries. This hampers their assessment and possible application in large-scale mass-rearing and open-field conditions. In contrast, the minimal alterations in the *LysRS* gene (precise single-base-pair changes) may fall under the SDN-2 class, as a small nucleotide template was supplied to repair the induced DSB. This classification is already deregulated in several countries (45). The *mini-LysRS* could be classified as cisgenic, as it was assembled entirely from endogenous sequences.

From a translation perspective, the identification of *LysRS* — a highly conserved, penetrant, early-development, and externally inducible marker — combined with our minimally invasive, nontransgenic approach to establish and rescue the mutant phenotype, provides a blueprint for developing *tsl*-based sexing strategies in other species. It also marks a significant step toward supporting sustainable pest management practices and reducing the burden of pest insects, along with the pathogens and parasites they transmit, on global healthcare systems and agriculture.

## Materials and Methods

The sequences of all oligonucleotides used in this study are provided in the *SI Appendix*, Table S1.

### Medfly Strains.

Medfly strains were obtained from the Insect Pest Control Laboratory in Seibersdorf (IPCL). The EgII laboratory WT strain (IPCL internal strain ID EgII_FF26) was used as a control, for injections, and for backcrosses. The homozygous *wp^–^*/*tsl^–^* strain (ICPL internal ID wp/tsl_FF21), which already has the target mutation in the *LysRS* gene (*LysRS_H>Y^ nat/nat^*), was used as a control and for crosses. EgII is a temperature-resistant strain and *wp^–^*/*tsl^–^* is a temperature-sensitive strain (24). The chromosome 5 balancer strain 68B (33) was used for crosses. Strains D53 (homozygous *wp^–^*/*tsl^–^* mutant with D53 inversion), *wp^–^*/*tsl^–^,* Benakeion and EgII (WT strains), GSS VIENNA-7, VIENNA-8^D53+^, and VIENNA-8 *Sr^2^* were used to generate sequencing data. Rearing conditions are detailed in the SI Materials and Methods section.

### In Silico Analysis of *tsl* Candidate Genes.

All FUN-annotated genes within the *tsl* genomic region (23) containing nonsynonymous mutations were analyzed in silico to narrow down the list of *tsl* candidates. Following the procedures outlined by Sollazzo et al. (23), transcriptomic data from *C. capitata* Benakeion and *wp^–^*/*tsl^–^* strains (BioProject No PRJEB57574) were assembled and mapped to the FUN-annotated EgII-3.2.1 genome (GCA_905071925.1) and manually inspected for polymorphisms using the Integrative Genomics Viewer v2.6 (IGV) (53). The coding sequence of each FUN-annotated gene with nonsynonymous mutations was used to screen the NCBI nr database using blastn v2.13.0 + (organism: *C. capitata*) (54) to retrieve the accession numbers and sequences of the genes and proteins in the NCBI reference genome Ccap_2.1 (GCA_000347755.4; Annotation release 103) (55). The protein sequences were used as queries to screen the NCBI nr database using blastp v2.13.0 + (organism: Diptera), to identify orthologs in other insects (56). These were used to create multi-species alignments using Geneious Prime (v2021.2.2) (57) and thus identify conserved domains in the protein sequences. Protein conservation in different species was determined using OrthoDB (v11) (58). If the candidate mutation was within a conserved domain, the effect of the amino acid change on the protein was further predicted using the SNAP2 classifier in PredictProtein (59). We also retrieved the expression profiles of the *Drosophila melanogaster* orthologs (flybase.org) to make an assessment for expression patterns and potential maternal deposition in medfly and conducted a literature review to investigate the functions of the candidate genes.

### Molecular Characterization of the *Cc_LysRS* Gene.

To confirm the predicted exon/intron structure and alternative splicing of the *C. capitata LysRS* gene (NCBI ID LOC101451416), RNA was extracted from WT EgII male and female flies (four each), a single prepupa, and embryos of different ages (0 to 1 h, 0 to 2 h, 2 to 4 h, 4 to 6 h, 26 to 28 h; 150 embryos each), transcribed to cDNA, and analyzed *via* PCR and RACE PCR. Details are given in the SI Materials and Methods. To confirm whether the H > Y candidate mutation matches the expected pattern of a *tsl* mutation, *LysRS* was sequenced in WT (EgII, Benakeion), GSS (VIENNA-7, VIENNA-8^D53+^, and VIENNA-8 *Sr^2^*) and *tsl* mutant strains (D53, *wp^–^*/*tsl^–^*), using Sanger sequencing or Next Generation Sequencing (NGS). For Sanger sequencing, genomic DNA was individually extracted from five to ten adult flies per strain. *LysRS* was PCR-amplified using primers P2087/2069, purified using the Zymo Clean & Concentrator-5 kit, and sent to Macrogen for sequencing. For NGS, genomic DNA was extracted from 40 adult flies (1:1 sex ratio, except for VIENNA-8 *Sr^2^* which included only males) for each strain using Blood & Cell Culture DNA Midi Kit (QIAGEN GmbH, Germany) following the manufacturer’s protocol. Sequencing libraries were prepared using the Nextera XT DNA Library Prep Kit Reference Guide (15031942 v03) and sequenced on an Illumina Nextera XT platform, resulting in 150-bp paired-end reads (Macrogen, Europe). Raw fastq NGS reads were imported in Geneious Prime 2023.0.2 and subjected to trimming using the BBDuk Trimmer plug-in (Version 1.0) with default settings. Trimmed reads were mapped to the *C. capitata LysRS* gene (NCBI Gene ID: LOC101451416) using the Bowtie2 plug-in (60) (Version 7.2.2 with default parameters: end-to-end alignment; high sensitivity).

### Cytogenetic Analysis of Medfly *LysRS*.

Salivary glands from third-instar medfly larvae were used to prepare polytene chromosomes for in situ hybridization in duplicate (10 nuclei per sample) as previously described (13, 23, 61, 62). Medfly salivary gland chromosome maps were used to identify hybridization sites (63).

### CRISPR/Cas9 Gene Editing to Generate the *LysRS_H>Y* Mutation.

The H > Y point mutation was introduced into the fifth exon of the medfly *LysRS* gene by CRISPR/Cas9 HDR-mediated gene editing using a single gRNA and a ssODN repair template. Suitable gRNA spacer sequences were evaluated in silico using Geneious Prime. The gRNA_LysRS target sequence (5′-TTAATCCTGGCTCACTACGA-3′) had zero off-targets in *C. capitata* genomes GCA_000347755.4 and GCA_905071925.1, and an on-target activity score of 0.503 (64). The gRNA was synthesized from a PCR-amplified double-stranded DNA template (P369 and P2091) by in vitro transcription as previously described (13). DNA and RNA quality and concentration were assessed using a Tekan Spark plate reader with a NanoQuant plate.

The 140-bp ssODN_LysRS_H>Y was designed with a point mutation (C > T) to introduce the desired histidine to tyrosine amino acid exchange (H516Y in XP_004536422 and H464Y in XP_004536423) and to destroy the PAM in the repair template to avoid reediting. Additionally, two silent point mutations, creating an MspI restriction site, were added to facilitate molecular analysis (Fig. 1). The single-stranded DNA repair template (sense orientation) was ordered as an Ultramer (P2090) from Integrated DNA Technologies and was dissolved in double-distilled water before use.

Embryonic microinjection mixes were prepared as previously described (34) using preassembled gRNA-Cas9 RNP complexes comprising 360 ng/µL Cas9 protein (PNA Bio Inc, CP01) incubated with 200 ng/µL gRNA_LysRS for 10 min at 37 °C, 200 ng/µL ssODN_LysRS_H>Y, and an end concentration of 300 mM KCl. EgII embryos were injected as previously described (13). Injected G_0_ embryos were kept in an oxygen chamber (max. 2 psi) at 19 °C until first-instar larvae began to hatch. Larvae were collected from the oil, transferred to larval food, and reared to adulthood at 25 °C.

### Identification and Molecular Analysis of *LysRS_H>Y* Mutants.

To identify G_0_ crosses with positive offspring, 100 G_1_ embryos were randomly collected from each G_0_ cross. Genomic DNA was extracted from each pool and the *LysRS* target region was amplified (P2087/P2069) using Phusion Flash High-Fidelity PCR Master Mix (Thermo Fisher Scientific) and the following profile: denaturation at 98 °C for 10 s followed by 30 cycles of 98 °C for 1 s, 57 °C for 5 s and 72 °C for 15 s, and a final elongation step at 72 °C for 1 min. We then mixed 10 µl of the reaction products with 1 µL MspI (20,000 units/ml; NEB), 2 µL 10× rCutSmart buffer (NEB) and 7 µL double-distilled water and incubated at 37 °C for 15 min. The products were analyzed by 2% agarose gel electrophoresis. The WT *LysRS* sequence amplicon contains one MspI restriction site, whereas the CRISPR mutated sequence amplicon contains two. Therefore, the 823-bp PCR product was expected to yield either two fragments (520 and 303 bp) or three fragments (450, 303, and 70 bp), depending on the genotype.

The same restriction assay was used for nonlethal genotyping to identify heterozygous and homozygous mutant flies in subsequent generations, as detailed in the *SI Appendix, Material and Methods*.

### Generation of Homozygous *LysRS_H>Y* Mutant Strains Using Three Independent Strategies.

Detailed information on the crosses implemented to establish homozygous *LysRS_H>Y^CRISPR^* mutant strains are given in the *SI Appendix*, Supporting Text S2, and all crosses for generations G_0_–G_20_, including the number of flies, genotypes, average hatching rates, and rearing temperatures, are summarized in *SI Appendix*, Fig. S6. In short, HDR^+^ heterozygous *LysRS_H>Y^ CRISPR^* G_1_ flies were inbred, and homozygous G_2_ mutants (*LysRS_H>Y^ CRISPR/CRISPR^*) were used to set up inbreeding and outcross cages. As homozygous mutant inbreeding at 25 °C initially resulted in low larval hatching, we pursued three independent strategies to ensure the survival of the strain: i) crossing the *LysRS_H>Y^ CRISPR^* mutation with the chromosome 5 balancer strain 68B carrying the *Sergeant-2* mutation on the balancer chromosome (33, 65), ii) alternating inbreeding of heterozygous mutants and outcrossing the resulting homozygous mutants to the EgII WT strain, and iii) introducing the *LysRS_H>Y^CRISPR^* mutation into the *wp^–^*/*tsl^–^* genomic background.

### Fertility and Fecundity Assessment.

To assess the fertility of homozygous *LysRS_H>Y^CRISPR^[E]* mutants and EgII WT flies at 23 °C, eggs (*n* = 3 × 100) were collected daily for 16 consecutive days. Larval hatching rates were calculated by subtracting the number of unhatched eggs from the collected embryos. Pupal and adult recovery rates were calculated by dividing the number of pupae or adults by the number of collected embryos. Strain fecundity was measured by counting the number of eggs laid per female per day. Eleven isofemale crosses (one female, three males) were set up per strain (*LysRS_H>Y^ CRISPR^[E]* mutants and EgII WT), and the number of eggs was counted every 24 h for 35 d.

### Temperature Sensitivity Test.

The tsl test was carried out at mid-embryonic developmental stages (age 24 to 29 h, “zygotic treatment”) as previously described (16, 24) and at preblastodermal stages (age 2 to 7 h, “maternal treatment”) to test for a maternal effect. Specifically, eggs were collected over a period of 5 h (9 am to 2 pm) from adults that started to oviposit 5 to 7 d before (180 adults per cage). If possible, 300 eggs were collected from each cage for each condition [i.e., control, treatment at preblastodermal stage (“maternal”), treatment at mid-embryonic developmental stage (“zygotic”)] to achieve three replicates each with 100 eggs (*n* = 3 × 100). Eggs were sieved from the water-filled collection tray, transferred to black filter paper strips, counted (100 eggs/strip), and placed onto 50 g of the carrot gel diet in a 9-cm Petri dish. For the control, plates were kept at 23 °C. To test for a maternal effect, eggs were counted at 23 °C and subsequently incubated at 34 °C with 70% RH for 24 h. For the zygotic-stage treatment, eggs were counted, kept at 23 °C for 24 h, and switched to 25, 28, 30, 31, 32, 33, or 34 °C (depending on the experimental setup) with 70% RH for 24 h. The eggs were placed in a Binder KT170 (E6.1) model incubator containing a water-filled tray to achieve 70% RH during incubation. The temperature and humidity were monitored using an EL-USB-2 data logger (Lascar Electronics). After treatment, Petri dishes with eggs were kept at 23 °C. Larval hatching was calculated for each replicate by subtracting the number of unhatched eggs from the number of collected embryos 5 d after egg collection. Pupal and adult recovery was calculated by dividing the number of pupae or adults by the number of collected embryos. SigmaPlot v14 was used to test for normality (Shapiro–Wilk) and for one-way ANOVA (Holm–Šidák test) to determine whether differences between the control and treatment groups were significant (*P* < 0.05).

### *LysRS* Minigene and piggyBac-Mediated Germline Transformation.

Vector *M6643* (*pXLBacII_mini-LysRS_IE1hr5-DsRed.T3-SV40*) contained a *LysRS* minigene (*mini-LysRS*) and a DsRed marker and was constructed by Gibson assembly. Therefore, the putative endogenous promoter region (252 bp upstream of the *LysRS* 5′ UTR) and the first 990 bp of the *LysRS* gene, including the alternatively spliced exons 1a/1b, the first introns, and part of exon 2 (Figs. 1*A* and 3*A*), were amplified from the genomic DNA. The remaining *LysRS* CDS, including the RACE-verified 3′ UTR, was amplified from cDNA. *LysRS* amplicons and the DsRed marker gene were ligated into the HindIII-digested *piggyBac* transformation vector *AH465* (5,997 bp) (*pXLBacII_IE1hr5-DsRed.T3-SV40* (44), kindly provided by A. Handler) by Gibson assembly, with the *mini-LysRS* and *IE1hr5-DsRed.T3* genes facing in opposite directions to prevent *mini-LysRS* transcription triggered by the *IE1hr5* promoter (Fig. 3*A*). The resulting plasmid (*M6643*) was used to transform chemically competent XL1blue *Escherichia coli* cells, sequenced, and purified. Details on the cloning procedure are given in the *SI Appendix, Material and Methods*.

*LysRS_H>Y^ CRISPR^[E]* embryos were injected with 300 ng/µl *M6643* (*pXLBacII_mini-LysRS_IE1hr5-DsRed.T3-SV40*) and 500 ng/µL insect codon-optimized hyperactive *piggyBac* transposase *Dm-^i^hyPBase* (*pSLfa_hsp70P-iPB7-hs3UTR_fa* (66), kindly provided by E. A. Wimmer) in embryonic injection buffer (5 mM KCl, 0.1 mM NaPO_4_, pH 6.8) as previously described (13). The resulting strains were kept at 23 °C until generation G_30_, and then transferred to 25 °C.

### Generation and Molecular Analysis of Homozygous *Mini-LysRS* Mutants.

G_0_ adults transiently expressing DsRed were backcrossed to the *LysRS_H>Y^ CRISPR^[E]* strain individually or in groups, whereas those lacking DsRed were crossed to the parental strain in groups. G_1_ individuals with DsRed fluorescence were again individually crossed to the *LysRS_H>Y^ CRISPR^[E]* strain and subsequently inbred to establish homozygous strains. Fluorescence microscopy was used to screen for heterozygous and homozygous mutant flies, using a DsRed filter (excitation 530 to 560 nm, emission 590 to 650 nm).

The *piggyBac* copy number in *mini-LysRS rescue* mutant strains was determined by ddPCR using DsRed as an indicator for the construct, and medfly *His3* (LOC101459256, encoding histone H3.3) as a reference housekeeping gene. iPCR was used to determine the genomic position of the *M6643* construct in the medfly genome. Details on ddPCR, iPCR, and image acquisition are given in the *SI Appendix*, *Material and Methods*. 

## Supplementary Material

Appendix 01 (PDF)

## Data Availability

*C. capitata LysRS_H>Y^CRISPR^* and *mini-LysRS rescue* mutant strains can be acquired from MFS. All data are included in the manuscript and/or *SI Appendix*.
