# Supplementary material for: Decoding and engineering temperature-sensitive lethality in Ceratitis capitata for pest control
Source: Proc Natl Acad Sci U S A. 2025 Jul 7;122(28):e2503604122. doi: 10.1073/pnas.2503604122 (PMC12280921; doi:10.1073/pnas.2503604122)
Supplement: Supplementary file 1 — Appendix 01 (PDF) [file pnas.2503604122.sapp.pdf]

PNAS

Supporting Information for

Decoding and engineering temperature-sensitive lethality in *Ceratitis capitata* for pest control

Roswitha A. Aumann, Georgia Gouvi, Maria-Eleni Gregoriou, Tanja Rehling, Germano Sollazzo, Kostas Bourtzis*, Marc F. Schetelig*

* Corresponding authors: Marc F. Schetelig and Kostas Bourtzis
Email: marc.schetelig@agrar.uni-giessen.de, k.bourtzis@iaea.org

This PDF file includes:

Supporting Material and Methods
Supporting text S1 to S3
Figures S1 to S14
Table S1
SI References

Supporting Materials and Methods

Medfly rearing conditions

All *Ceratitis capitata* strains were maintained either at 25±1°C with 48% relative humidity (RH) and a 14-h photoperiod or at 23±1°C, with 40–60% RH and 24 h illumination. Adults were provided with a 3:1 (v/v) mixture of sugar and enzymatic yeast hydrolysate, and water *ad libitum*. Larvae were provided with a gel diet made up as follows: 1.4 kg frozen carrots (Demeter-Felderzeugnisse GmbH, 601701) cooked in 1 l distilled water (dH_2O), 500 g carrot powder (Van Drunen Farms, 35824-A02), 168 g enzymatic yeast hydrolysate (MP Bio), 20 ml 25% HCl (Rotipuran, Cas 7647-01-0), and 16 g sodium benzoate (VWR, Cas 532-32-1), topped up to 1 L with dH_2O. CO_2 was used to anesthetize adult files for screening and sexing.

Molecular characterization of the **Cc_LysRS** *gene*

To confirm the predicted gene structure and isoforms of the *C. capitata LysRS* gene (NCBI ID LOC101451416), the Monarch total RNA miniprep kit (NEB) was used to extract RNA from WT EgII male and female flies (four each), a single pre-pupa, and embryos of different ages (0–1 h, 0–2 h, 2–4 h, 4–6 h, 26–28 h; 150 embryos each). We prepared cDNA from 1 µg total RNA using the iScript gDNA clear synthesis kit (Bio-Rad). Oligonucleotides P2068, P2242, P2070 and P2069 were used with Phusion Flash polymerase to amplify the different isoforms on a Bio-Rad C1000 Touch thermal cycler, with the following profile: denaturation at 98°C for 10 s, followed by 35 cycles of 98°C for 1 s, 56°C for 5 s and 72°C for 45 s, and a final elongation step at 72°C for 1 min. We used the NEBNext poly(A) mRNA magnetic isolation module (NEB) to isolate $polyA^+$ RNA from the pre-pupa total RNA. This was used to prepare full-length cDNA for RACE using the SMARTer RACE 5′/3′ kit (Takara) with the gene-specific oligonucleotides P2244 and P2243.

Molecular analysis of **LysRS_H>Y** *mutants (non-lethal genotyping)*

A non-lethal genotyping assay was used to identify heterozygous and homozygous *LysRS_H>Y* mutant flies before setting up crosses. Genomic DNA was isolated from a single leg according to the Platinum Direct PCR Universal Master Mix (Invitrogen) lysis protocol. In detail, flies were anesthetized, and one middle leg was removed and digested with 0.6 µl proteinase K in 20 µl lysis buffer for at least 1 min. The reaction was stopped by heating to 98°C for 1 min, and 1 µl of the supernatant was used as template in a 15-µl PCR comprising 2× Platinum Direct PCR Universal Master Mix (Invitrogen), oligonucleotides P2087 and P2069, and double-distilled water to top up. The following amplification profile was used: denaturation at 94°C for 2 min followed by 35 cycles of 94°C for 15 s, 60°C for 15 s and 68°C for 20 s. We used 10 µl of the reaction products for the MspI assay and 2% agarose gel electrophoresis as described above. The genotype of the G_1 flies in the initial crosses was confirmed by sequencing the PCR product (Macrogen, with oligonucleotide P2087) following cleanup using the Zymo Clean & Concentrator-5 kit.

Construction of the **LysRS** *minigene plasmid*

Vector *M6643* (*pXLBacII_mini-LysRS_IE1hr5-DsRed.T3-SV40*) contained a *LysRS* minigene (*mini-LysRS*) and a DsRed marker and was constructed by Gibson assembly. The putative endogenous promoter region (252 bp upstream of the *LysRS* 5′ UTR) and the first 990 bp of the *LysRS* gene, including the alternatively spliced exons 1a/1b, the first introns, and part of the exon 2 (Figs 1a and 3a), was amplified from the genomic DNA of a single EgII female fly using the primers P2268/P2270 and Phusion Flash High-Fidelity PCR Master Mix in a 35-µl reaction. The following amplification profile was used: denaturation at 98°C for 10 s followed by 30 cycles of 98°C

for 1 s, 56°C for 5 s and 72°C for 15 s, and a final extension step at 72°C for 1 min. The remaining *LysRS* CDS, including the RACE-verified 3′ UTR, was amplified from cDNA (1:10 dilution) prepared from an EgII female fly total RNA pool using primers P2267/P2269 as described above, except the annealing temperature in each cycle was reduced to 48°C. The products (1,289 bp genomic DNA and 1,638 bp cDNA, including Gibson cloning overhangs) were separated by agarose gel electrophoresis, purified using the Zymoclean Gel DNA recovery kit and eluted in 9 µl TE buffer.

The *piggyBac* transformation vector *AH465* (*pXLBacII_IE1hr5-DsRed.T3-SV40* (1), kindly provided by A. Handler) was digested with HindIII and dephosphorylated with Antarctic phosphatase for 30 min at 37°C. After deactivation for 2 min at 80°C, the *LysRS* amplicons were ligated into the vector (5,997 bp) by Gibson assembly with the *mini-LysRS* and *IE1hr5-DsRed.T3* genes facing in opposite directors to prevent *mini-LysRS* transcription triggered by the *IE1hr5* promoter (Fig. 3a). The resulting plasmid (*M6643*) was used to transform chemically competent XL1blue *Escherichia coli* cells, and the correct sequence was confirmed. Endotoxin-free plasmid DNA was purified using the NucleoBond Xtra Maxi EF kit (Machery-Nagel).

LysRS_H>Y^{CRISPR}*[E]* embryos were injected with 300 ng/µl *M6643* (*pXLBacII_mini-LysRS_IE1hr5-DsRed.T3-SV40*) and 500 ng/µl insect codon-optimized hyperactive *piggyBac* transposase *Dm-*^{i}*hyPBase* (*pSLfa_hsp70P-iPB7-hs3UTR_fa* (2), kindly provided by E. A. Wimmer) in embryonic injection buffer (5 mM KCl, 0.1 mM $NaPO_4$, pH 6.8) as previously described (3). The resulting strains were kept at 23°C during all development stages.

Analysis of* mini-LysRS *strains: Digital droplet PCR and inverse PCR

Digital droplet PCR (ddPCR) was used to determine the *piggyBac* copy number in *mini-LysRS rescue* mutant strains using DsRed as an indicator for the construct, and medfly *His3* (LOC101459256, encoding histone H3.3) as a reference housekeeping gene. Genomic DNA from heterozygous *mini-LysRS rescue* mutants was mixed with ddPCR 2× Supermix for probes (no dUTP, Bio-Rad), DsRed-FAM primer/probe-mix (containing oligonucleotides P49, P50, and probe DsRed-FAM), CcHis3-HEX primer/probe-mix (containing oligonucleotides P101, P103, and probe CcHis-HEX) and EcoRI to a final concentration of 1x Supermix, 900 nM oligonucleotides, 250 nM probes and 2 U EcoRI in a total volume of 25 µl in a ddPCR 96-well plate (Bio-Rad). The plates were sealed with pierceable foil using a PX1 PCR plate sealer (Bio-Rad). Droplets were generated using automated droplet generation oil for probes and DG32 automated droplet generator cartridges in an automated droplet generator AutoDG equipped with pipet tips for the AutoDG system (Bio-Rad). The plates containing the generated droplets were sealed as previously described and thermal cycling was done in a deep well block on a Bio-Rad C1000 Touch Thermal Cycler. Enzyme was activated at 95°C for 10 min followed by 40 cycles of 94°C for 30 s and 55°C for 1 min. Enzyme was deactivated at 98°C for 10 min. Fluorescence of droplets was analyzed by the QX200 droplet reader with ddPCR droplet reader oil and data were analyzed using QuantaSoft (Regulatory Edition, Bio-Rad).

To determine the genomic position of the *M6643* construct, genomic DNA from heterozygous individuals was amplified by inverse PCR (iPCR). DNA was digested with MspI for 1 h at 37°C, then precipitated with 3 M sodium acetate in cold ethanol and ligated overnight at 16°C using T4 DNA ligase (NEB). Following another round of precipitation, 5′pBac iPCR was carried out with oligonucleotides mfs11/mfs10, 3 µl ligated DNA, and Phusion Flash High-Fidelity PCR Master Mix in 20-µl reactions with the following amplification profile: denaturation at 98°C for 10 s, followed by five cycles of 98°C for 1 s, touchdown 66–56°C (–2°C per cycle) for 5 s, and 72°C for 1 min, then 30 cycles of 98°C for 1 s, 56°C for 5 s and 72°C for 1 min, with a final 72°C extension step for 1 min. Similarly, the 3′pBac integration site was determined using oligonucleotides mfs34/P115 under the same reaction conditions, except the touchdown phase in the first set of cycles was 60–

50°C and the annealing temperature in the second set of cycles was 50°C. Products were purified from agarose gels and sequenced as described above.

Image acquisition

Flies were anesthetized with CO_2 and cooled on ice to facilitate image acquisition. A Leica M205FC stereo microscope (PLANAPO 1.0× objective), DFC7000 T camera and Application Suite X software (LAS X v3.7.2.22383) were used for bright-field and fluorescence imaging (DsRed filter, excitation 530–560 nm, emission 590–650 nm). Images were processed with Adobe Photoshop Lightroom v6.0 to enhance brightness and contrast.

Supporting Text S1

***Selection and analysis of the* tsl *candidate gene* LysRS**

Wild-type, GSS, and *tsl* mutant strains of *C. capitata* have previously been analyzed using genomic, transcriptomic, bioinformatic, and cytogenetic techniques to identify the so-called *tsl* genomic region (4). This region is located on the right arm of chromosome 5, between the *white pupae* (*wp*) and *glucose-6-phosphate 1-dehydrogenase* (*Zw*) loci, spanning from 59B to 61C of the trichogen polytene chromosome map, which most likely corresponds to region 76B-79C of the salivary gland polytene chromosome map (4). We screened all genes within this region that harbor nonsynonymous mutations, considering them as potential *tsl* candidates. In addition to the screening process described by Sollazzo et al. (2022), which analyzed transcripts of *C. capitata* Benakeion and wp^{-}/tsl^{-} strains mapped to the EgII-3.2.1 genome (GCA_905071925.1) and identified, among others, the *tsl* candidate gene *deep orange* (*dor*) (5), we conducted further analyses. First, to evaluate the concordance between the two published *C. capitata* genome versions and their respective annotations, we searched the NCBI reference genome Ccap_2.1 (GCA_000347755.4 (6); Annotation release 103) for each FUN-annotated candidate gene from the EgII-3.2.1 genome (GCA_905071925.1 (3)). Assembled transcripts of *C. capitata* Benakeion and wp^{-}/tsl^{-} strains (4) were then mapped to the retrieved annotated Ccap_2.1 gene sequences and again manually inspected for polymorphisms. Subsequently, candidate genes were further refined based on knowledge derived from earlier studies based on deletion and transposition mapping, suggesting that the *tsl* gene should be positioned outside the pericentric D53 inversion (spanning 69C-76B on the salivary gland polytene chromosome map) and near to the (molecularly unidentified) *Sergeant-2* (Sr^2) gene (78B on the salivary gland polytene chromosome map) (3, 4, 7-9), and on the assumption that a valid candidate gene should be conserved among insects, have essential functions, and harbor a mutation in a critical region, such as a domain, motif, binding site or active site with a strong predicted impact on protein function. Additionally, expression profiles of *Drosophila melanogaster* orthologs were analyzed, assuming that expression patterns in medfly would be comparable. A valid *tsl* candidate gene was expected to be ubiquitously and continuously expressed, reflecting the presence of the tsl phenotype in all developmental stages, with particularly high expression in ovaries and early embryonic stages, indicating maternal deposition and potentially the known maternal effect. This screening process led to *LysRS* being chosen as the most promising candidate.

Notably, the comparison of the *LysRS* loci from the two published *C. capitata* genome versions (3, 6) showed only 97.8% identity and revealed 28 gaps scattered along the 2,875-bp sequence (25 missing and three additional nucleotides in EgII-3.2.1 GCA_905071925.1 compared to Ccap_2.1 GCA_000347755.4; Fig. S1). These gaps result in frameshifts that introduce premature stop codons, leading to an incorrectly-truncated gene and protein in the FUN-annotation (ID:FUN_017610, GCA_905071925.1). Based on the incorrect sequence in the EgII-3.2.1 genome

and the incorrectly-truncated FUN-annotation, *LysRS* was initially only identified as a "gene featuring nonsynonymous mutation", but not listed as a promising candidate by Sollazzo et al. (4). The (real) candidate mutation, which led us to choose *LysRS* as our top *tsl* candidate gene for this study, was only detectable in the Ccap_2.1 genome that uses an NCBI annotation (Fig. S1).

LysRS was sequenced in several WT, GSS and *tsl* mutant strains, and the H>Y candidate mutation was confirmed to match the expected pattern of a *tsl* mutation (homozygous 'H' in WT, homozygous 'Y' in *tsl* mutants and GSS females, heterozygous 'H/Y' in GSS males). To investigate a possible interaction between *LysRS* and the recently analyzed and nearby situated *tsl* gene *deep orange* (5), we also checked the *dor* allele in these strains. Interestingly, VIENNA-8 Sr^2 males, which display the WT phenotype as expected, were heterozygous for the *LysRS* H>Y mutation (as anticipated for the *tsl* gene), but homozygous for the mutant *dor* tsl^- allele. Specifically, all six SNPs in the *dor* gene show the wp^-/tsl^- nucleotides (described by Sollazzo et al., 2024: I83M, D100E, H313P, I458L, E839K, L940I). This data suggests that one copy of the *LysRS* WT allele can restore the WT phenotype in a homozygous *dor* mutant and strongly supports our conclusion that *LysRS* is the causal gene for the tsl phenotype (independent of *dor*).

Cytogenetic analysis revealed that *LysRS* is located in region 77A/B of the salivary gland polytene chromosome map (Fig. S4), one cytogenetic band apart from *wp* (76B (3)). On scaffold 5 of the EgII-3.2.1 genome (GCA_905071925.1), the distance from *LysRS* to *wp* is approximately 1.4 Mbp (including seven regions of unknown sequence, N-gaps). The distances from *LysRS* to the right breakpoint of the D53 inversion and *Zw* are approximately 1 Mbp (including six N-gaps) and 4.8 Mbp (including 20 N-gaps), respectively.

Supporting Text S2

Generation of homozygous LysRS *mutants*

To induce the *LysRS* candidate mutation in the temperature-resistant WT strain EgII, we injected 305 WT EgII embryos with a single-stranded oligodeoxynucleotide (ssODN) donor template, designed to introduce the candidate mutation and two silent mutations, a single guide RNA (gRNA) and Cas9 protein (Fig. 1c). Sixty-eight G_0 adults (33 males, 35 females) survived the injection and were backcrossed to the WT EgII strain individually or in small groups (six single crosses with one G_0 male crossed to five EgII females, six single crosses with one G_0 female crossed to three EgII females, 10 group crosses with 5–7 G_0 males or females, crossed to 5–10 EgII females or males, respectively).

Genotyping pools of G_1 embryos identified the female G_0 individual cross 'F2' as the most promising one to produce HDR^+ offspring (*LysRS_H>Y*^{CRISPR}). G_0-F2 produced 369 G_1 descendants, and 137 were analyzed by non-lethal genotyping, resulting in 11 male and 19 female HDR^+ heterozygous *LysRS_H>Y*^{CRISPR} G_1 flies. These flies were used to set up two inbreeding crosses (cage A, 11 females and five males; cage B, five females and two males) and one outcross cage (15 EgII females and two *LysRS_H>Y*^{CRISPR} males). Eggs from these crosses were collected three times, resulting in 1,960 pupae (inbreeding cage A) and 645 pupae (inbreeding cage B). Overall, 885 G_2 flies descended from one 48-h egg collection (inbreeding cage A) eclosed over a period of 7 days, and 218 of them were analyzed using the described MspI-based non-lethal genotyping assay. Only WT and heterozygous mutant flies were found during the first 3 days (day 1, 27 WT and 60 heterozygous; day 2, 18 WT and 38 heterozygous; day 3, three WT and seven heterozygous). All genotypes were found on later days (day 4, 12 WT, 12 heterozygous, 11 homozygous; day 5, two WT, two heterozygous and 15 homozygous; day 7, one WT, one heterozygous and nine homozygous). This proportional distribution of different genotypes (WT, heterozygous, and

homozygous *LysRS_H>Y*^{CRISPR} mutants) over the adult eclosion period suggested a slow development phenotype in homozygous mutant flies. The delay was caused by slower development during the larval stage, a phenotype that was also observed in subsequent generations to some extent (1–2 days delay compared to the WT strain). However, in later generations (G_{15} onwards), the development of the *LysRS_H>Y*^{CRISPR} homozygous mutant was comparable to WT EgII flies at all stages (≤ 1 day delay).

G_2 offspring of inbred heterozygous mutant G_1 flies were analyzed by non-lethal genotyping. Homozygous G_2 mutants (*LysRS_H>Y*$^{CRISPR/CRISPR}$) were used to set up inbreeding and outcross cages (*LysRS_H>Y*^{CRISPR} inbreeding, 19 females and eight males; outcross, 20 EgII females and eight *LysRS_H>Y*^{CRISPR} males). Homozygous mutant inbreeding at 25°C resulted in low larval hatching, and the strain was lost in the next generation (G_3). Therefore, three independent strategies were pursued to ensure the survival of the strain: (i) crossing the *LysRS_H>Y*^{CRISPR} mutation with the chromosome 5 balancer strain 68B. This strain carries the homozygous lethal *Sergeant-2* (*Sr*^{2}) mutation on its balancer chromosome FiM1 (10, 11). The overlapping pericentric inversions of FiM1 suppress recombination throughout chromosome 5, enabling the maintenance of potentially lethal mutations (11). This ensured the presence of the *LysRS* mutation in heterozygous form and facilitated the identification of carriers, as individuals carrying the FiM1 chromosome and the *Sr*^{2} mutation exhibit three white stripes on the abdomen instead of two found on wild-type or *LysRS_H>Y*^{CRISPR} mutant flies; (ii) alternating inbreeding of heterozygous mutants and outcrossing the resulting homozygous mutants to the EgII WT strain, to finally establish a homozygous line carrying only the paternal genomic background (EgII); and (iii) introducing the *LysRS_H>Y*^{CRISPR} mutation in the *wp*$^-$/*tsl*$^-$ genomic background, to determine whether this genetic background is beneficial for the maintenance of the *LysRS* CRISPR mutation at 25°C.

For the first strategy, 15 heterozygous G_2 mutant females were crossed to five 68B males. Mutants carrying the *LysRS_H>Y*^{CRISPR} mutation and balancer chromosome were identified by non-lethal genotyping and screening for the Sergeant-2 phenotype (three white stripes on the abdomen). Inbreeding these mutants resulted in ~66% heterozygous *LysRS_H>Y*^{CRISPR} mutants carrying the balancer chromosome, identified by the Sergeant-2 phenotype, and ~33% homozygous *LysRS_H>Y*^{CRISPR} mutants, recognizable by their WT phenotype (two white stripes), which typically eclose 2–3 days later than the heterozygous mutants, and a stable larval hatching rate of 40–50% in the subsequent generations (25°C). Inbreeding the homozygous mutants from these crosses resulted in low fertility and fecundity at 25°C so a lower temperature (23°C) was chosen to rear the homozygous mutants (hatching rates ~70%, Fig. S6). This strain (*LysRS_H>Y*^{CRISPR}*[B]*) was used to produce the data in Figs S9 and S10.

For the second strategy, homozygous G_2 mutants were used to set up inbreeding and outcross cages. The inbreeding of homozygous mutants led to low fertility and fecundity at 25°C but inbreeding heterozygous mutants and outcrossing homozygous mutants to the EgII WT strain resulted in larval hatching rates of 67–91% (Fig. S6). Therefore, the strain was maintained at 25°C by alternating inbreeding of heterozygous and outcrossing of genotyped homozygous mutants until generation G_9, to ensure survival, refresh the genetic background, avoid an inbreeding bottleneck, and allow for the selection of spontaneous beneficial compensatory mutations. From generation G_{10} onwards, the strain (*LysRS_H>Y*^{CRISPR}*[E]*) was maintained in a homozygous state at 23°C (hatching rates ~80–90%, Fig. S6). This strain was used to produce the data in Figs 2 and 3, and Figs S7 and S8, and to generate the *minimal LysRS rescue* mutant strains. In generation G_{13}, part of the colony (160 pupae) was transferred to 25°C to test their fitness at this previously 'suboptimal' evaluated temperature. Adults eclosed from these pupae produced offspring with a 69% larval hatching rate but only a 24% adult eclosion rate (assessed embryos n = 3×100). In the next generation, maintained at 25°C throughout development, only 4% of the embryos hatched. In subsequent generations, larval hatching rates continued to fluctuate (Fig. S6), and multiple

attempts of sustained rearing at 25°C were required before the survival rates eventually stabilized. Homozygous *LysRS_H>Y^{CRISPR}[E]* mutants are now exhibit survival rates comparable to WT at the same temperature, achieving approximately 90% larval hatching and ~80% adult eclosion rates (Fig. S9). *LysRS_H>Y^{CRISPR}[E]* have been successfully reared at 23°C and 25°C for over 40 and 30 generations, respectively, with their tsl phenotype frequently confirmed through tsl tests.

For the third strategy, homozygous *LysRS_H>Y^{CRISPR}[E]* (*wp$^{+/+}$ LysRS_H>Y$^{CRISPR/CRISPR}$*) females (G_{11}) were crossed to homozygous *wp^{-}/tsl^{-}* males (*wp$^{-/-}$ LysRS_H>Y$^{nat/nat}$*) at 23°C. Heterozygous female offspring were crossed again to *wp^{-}/tsl^{-}* males to trigger recombination between the *wp^{-}* and *LysRS* loci. Individuals of the next generation were phenotyped for the *wp* mutation (white puparium) and genotyped for the CRISPR mutation (MspI digest) to detect *wp^{-} LysRS_H>Y^{CRISPR}* recombinants (*wp$^{-/-}$ LysRS_H>Y$^{nat/CRISPR}$*). These recombinants were inbred, and their offspring were genotyped to identify double homozygous mutants (*wp$^{-/-}$ LysRS_H>Y$^{CRISPR/CRISPR}$*; Fig. S6), hereafter described as *LysRS_H>Y^{CRISPR}[t]*. After three generations at 23°C, part of this colony (160 pupae) was transferred to the 'suboptimal' 25°C rearing temperature to determine whether the partial *wp^{-}/tsl^{-}* genomic background would influence the performance at this temperature. Adults emerging from these pupae produced offspring with an average 72% larval hatching rate, but the hatching rate in the next generation dropped to 20%. However, in contrast to the slow increase in the hatching rate of the *LysRS_H>Y^{CRISPR}[E]* mutants, the *LysRS_H>Y^{CRISPR}[t]* strain achieved hatching rates of 77% and 60% in the third and fourth generations, respectively (Fig. S6b). The strain has been successfully reared at 25°C for more than 35 generations, with its tsl phenotype repeatedly verified through testing.

All crosses for generations G_0–G_{20}, including the number of flies, genotypes, average hatching rates, and rearing temperatures, are summarized in Fig. S6.

Supporting Text S3

Rescue with a* LysRS *minigene

To engineer a minigene for the tsl phenotype rescue assay, we aimed to identify the endogenous minimal promoter of *LysRS*. Therefore, we compared the sequence upstream of *LysRS* in medfly and *D. melanogaster* (CG12141). In both species, the E3 ubiquitin-protein ligase gene *HECW2* is found immediately upstream of *LysRS* in reverse orientation (*D. melanogaster* CG42797, *C. capitata* LOC101451725). Accordingly, the putative promoter region in medfly was defined as the sequence between the annotated 5′ untranslated regions (5′ UTRs) of these neighboring genes (Fig. S12).

The 252-bp long putative promoter region (Fig. S12), the annotated 5′ UTR, exon 1a, 1b, and part of exon 2 of the *LysRS* gene were amplified from genomic DNA (i.e., including the first introns) to ensure that alternative splicing was preserved in the minigene. The remaining coding sequence was amplified from cDNA (without introns) to keep the minigene as short as possible (Fig. 3a). Sequence and length of the annotated 3′ UTR were verified by 3′ rapid amplification of cDNA ends (3′-RACE). The *mini-LysRS* sequence (2,854 bp) and a DsRed marker (2,033 bp) were then transferred to a *piggyBac* transformation vector (Fig. 3a) and injected, together with the *piggyBac* helper plasmid, into 809 *LysRS_H>Y^{CRISPR}[E]* embryos. We observed transient DsRed expression in 192 of 335 hatched larvae, 136 of which survived to adulthood (overall: 223 adults).

G_0 adults with transient DsRed expression were crossed to the parental *LysRS_H>Y^{CRISPR}[E]* strain either individually (10 single crosses) or in groups (10 group crosses, 11–13 G_0 individuals each). Those with no transient expression (38 females and 49 males) were crossed to the parental strain in groups. Nine crosses (one single cross, eight group crosses) produced G_1 offspring with DsRed fluorescence. Twelve G_1 individuals with different DsRed intensities were picked and individually crossed to the *LysRS_H>Y^{CRISPR}[E]* strain. Seven randomly picked families were analyzed by

droplet digital PCR (ddPCR) to determine the copy number of the *piggyBac* transformation vector. If only one copy was found, G_2 offspring were inbred (MG6_m1 (A), FG3_m1 (B), FG7_m5 (C) and FG1_m5 (D)). If more than one copy was found, G_2 offspring were individually crossed (FG3_m2 (E), FG3_f1 (F) and M1_m1 (I)). Finally, six families could be maintained (MG6_m1 (A), FG3_m1 (B), FG7_m5 (C), FG3_m2_m1 (E), FG3_f1_m1 (F) and M1_m1_m2 (I)) and were further analyzed.

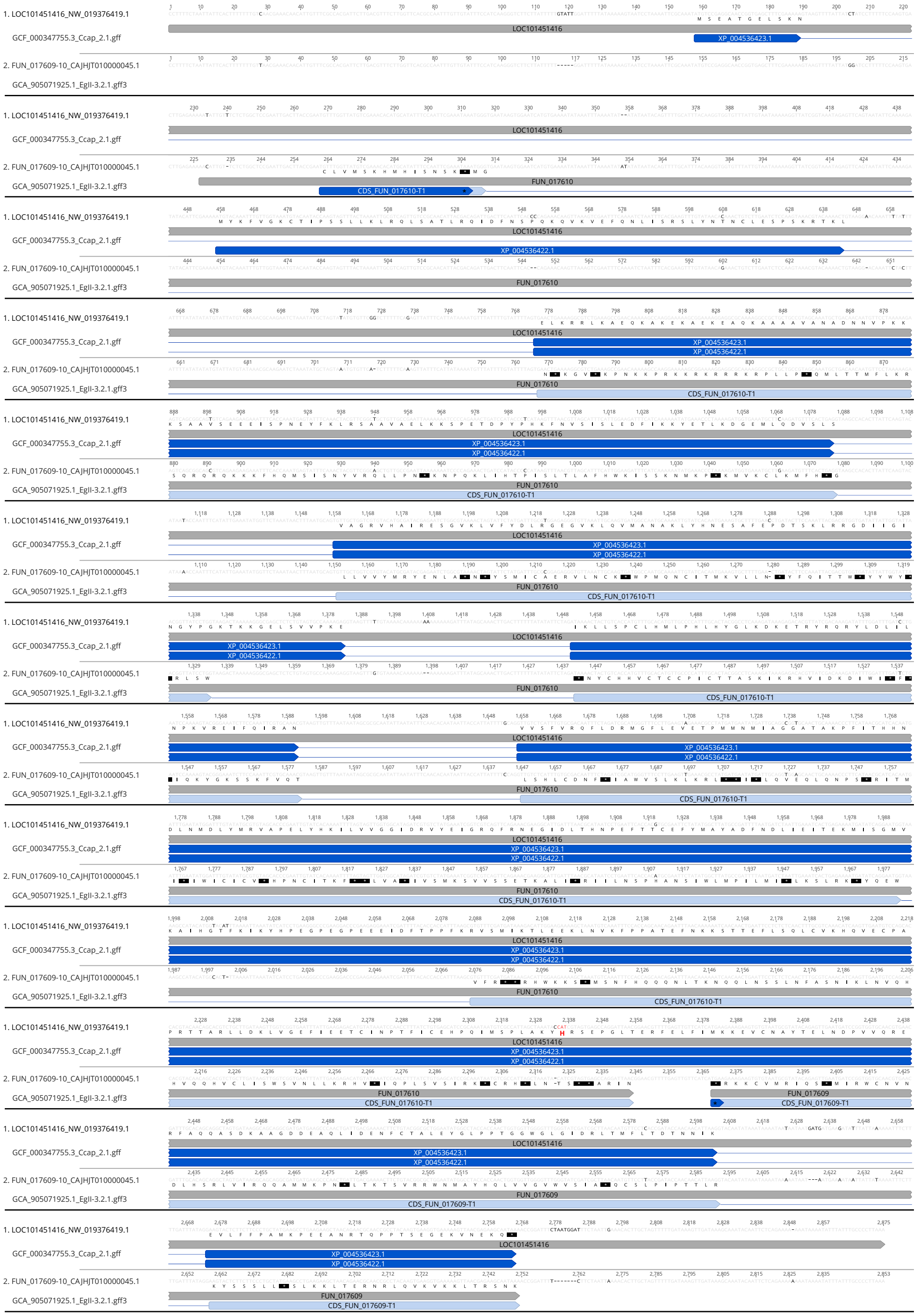

Fig. S1. | Annotated nucleotide sequence and translation (by annotation) of the *Lysine--tRNA ligase* gene in the two *C. capitata* genome assemblies, aligned using MUSCLE. Predicted genes are annotated in grey, coding sequences in blue (open reading frame (ORF): dark blue, lack of ORF: light blue). Disagreements in the nucleotide sequences and stop codons (*) in the amino acid sequence are highlighted in black, the *tsl* candidate mutation in red.

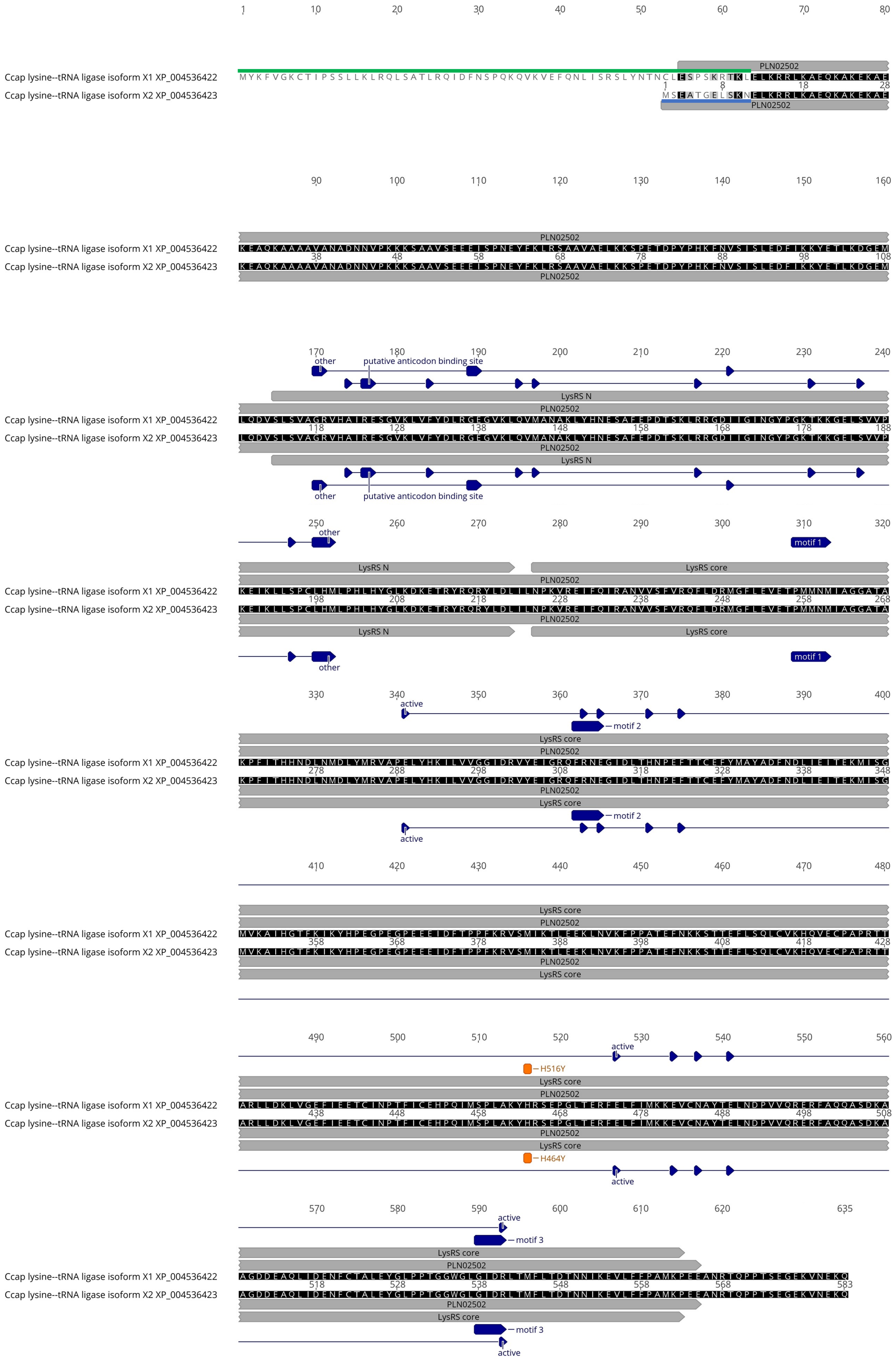

Fig. S2. | Amino acid sequence alignment of the two *C. capitata* Lysine--tRNA ligase protein isoforms using Clustal Omega. Alternative splicing of exons 1a (corresponding amino acids (aa) underlined in blue) and 1b (corresponding aa indicated in green) results in two transcripts, which are translated into two protein isoforms (X2 and X1, respectively). Identical residues are shown in black. Predicted motifs, active sites and the putative anticodon binding site are shown in blue. The PLN02502 and LysRS core domains are shown in grey. The position of the *tsl* candidate mutation is indicated in orange.

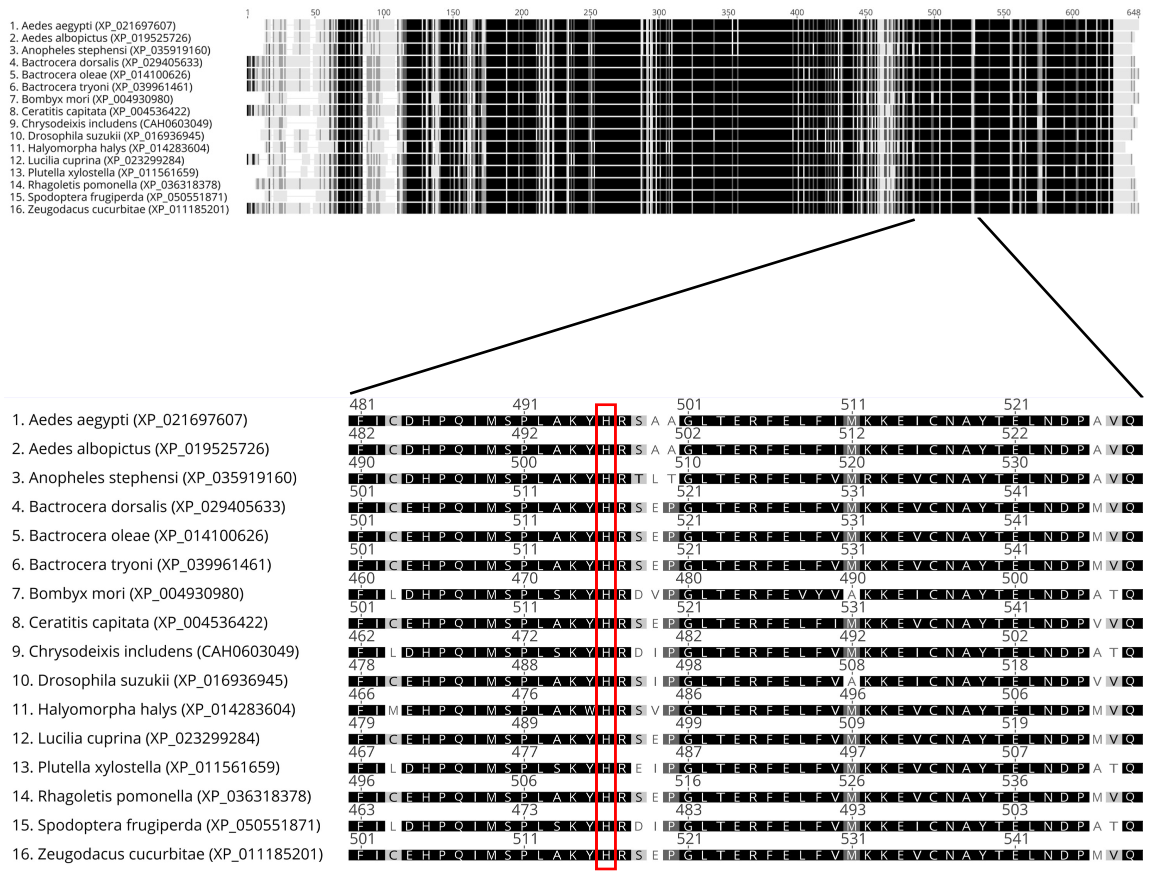

Fig. S3. | Amino acid sequences of Lysine--tRNA-ligase X1-isoform homologs from selected insect pests aligned using MUSCLE. Examples are shown representing all main groups of SIT target species, including plant pests (Bombycidae, Drosophilidae, Noctuidae, Plutellidae, Pentatomidae, Tephritidae), livestock pests (Calliphoridae), and human disease vectors (Culicidae). Identical residues are shaded in black and conserved residues in grey. The position and conservation of the *C. capitata tsl* candidate mutation (H516Y in XP_004536422) are shown in an enlarged section and highlighted in a red box.

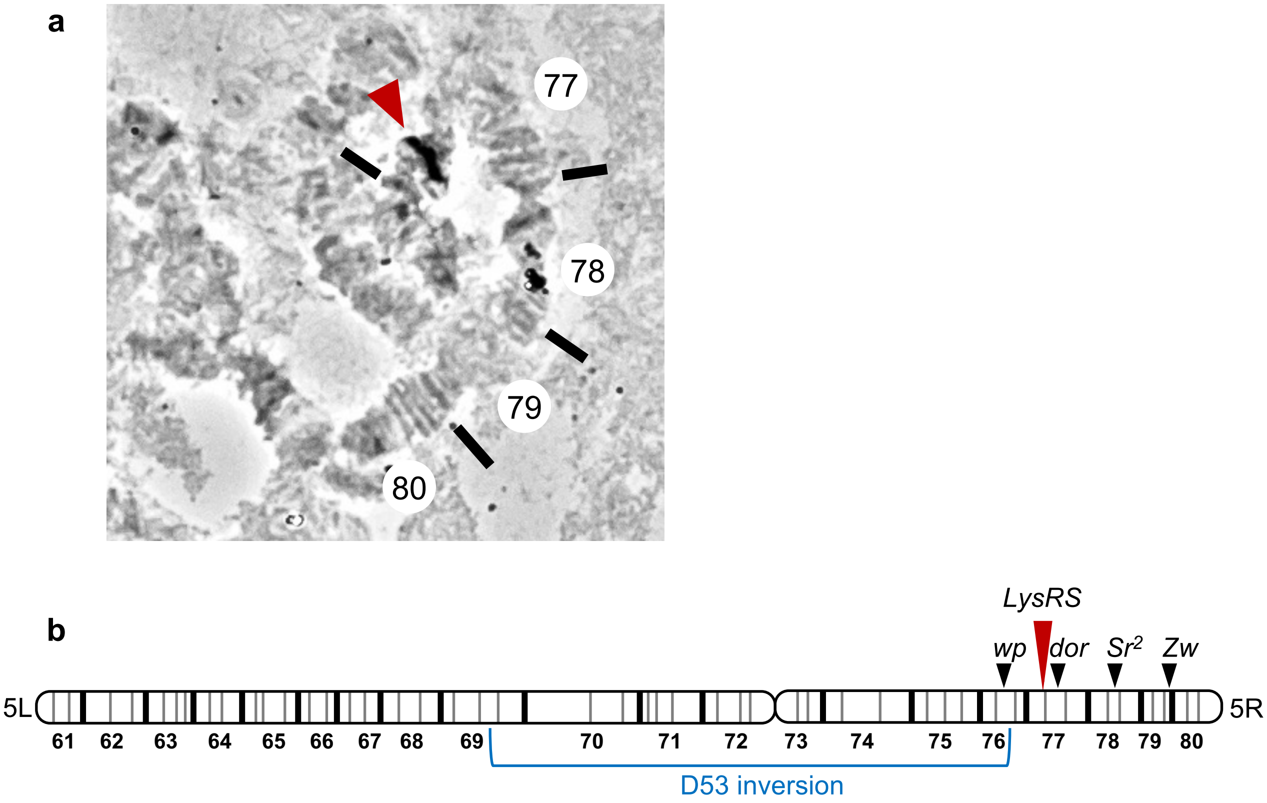

Fig. S4. | The genomic position of the *LysRS* gene. a, *In situ* hybridization of *C. capitata* polytene chromosome spread reveals the position of the *LysRS* gene on the right arm of chromosome 5, in segment 77A/B (red arrow in micrograph). Segments 77-80 are marked and numbered. **b,** Schematic overview of *C. capitata* chromosome 5 (5L = left arm, 5R = right arm), and the positions of *white pupae (wp,* 76B) (3)*, LysRS* (77A/B, this study, red arrow), *deep-orange* (*dor, 77B*) (8), *Sergeant-2* (*Sr^2,* 78B) (9) and *glucose-6-phosphate 1-dehydrogenase* (*Zw,* 79C) (10)*,* and the extent of the chromosomal inversion D53 (69C-76B; blue), which is used in the VIENNA-8^{D53+} GSS (3, 11). The position of *LysRS* coincides with the previously suggested region of the causal *tsl* gene (59B-61C of the trichogen polytene chromosome map, which most likely corresponds to region 76B-78C of the salivary gland polytene chromosome map). This region was circumscribed to be located on the right arm of chromosome 5, downstream of *wp* and near to the *Sergeant-2* gene, but outside the pericentric D53 inversion (4, 9, 11, 12).

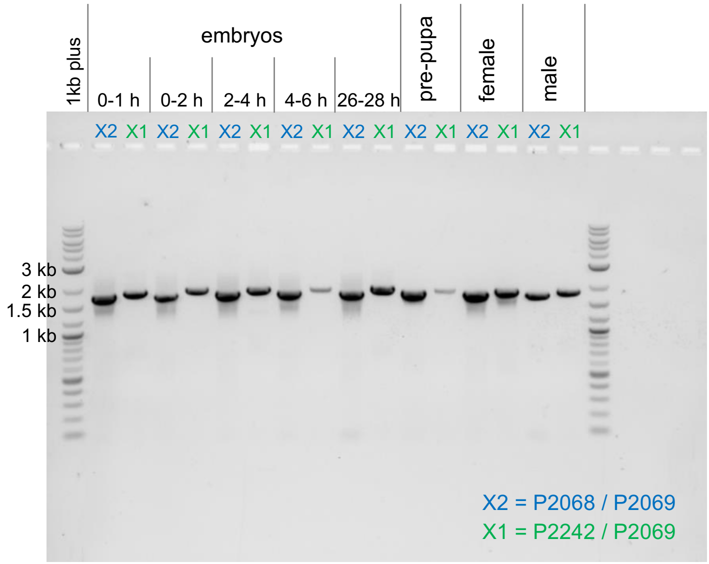

Fig. S5. | Verification of the alternative splicing variants in cDNA samples from different developmental stages of WT EglI. Uncropped version of the gel shown in Fig. 1b.

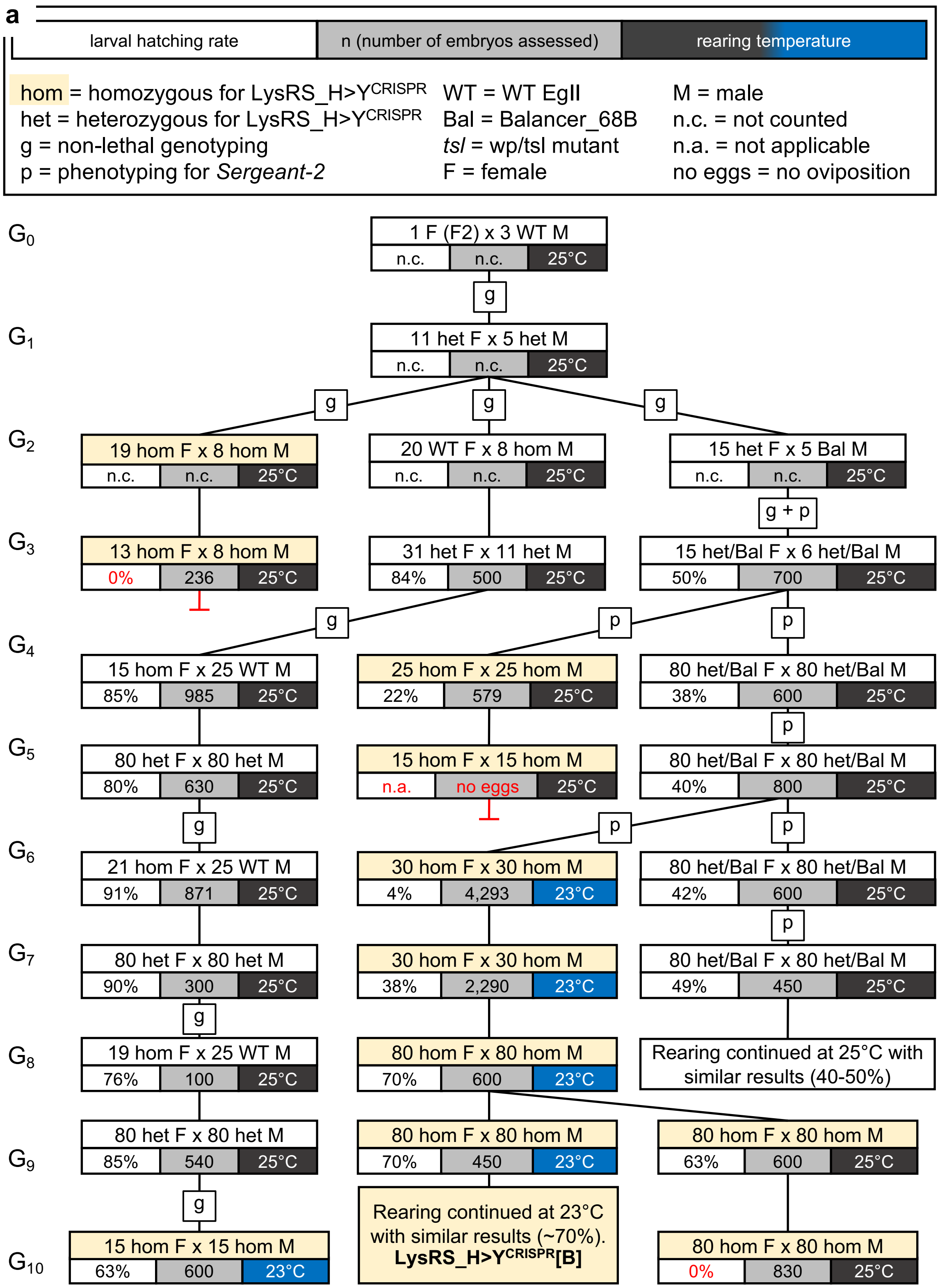

Fig. S6. | Generation and maintenance of homozygous *LysRS_H>Y^CRISPR* mutants. a, The cross setup for generations G_0-G_{10}, including the number of flies, genotypes, the average hatching rate, the number of embryos assessed to calculate it, and the rearing temperature.

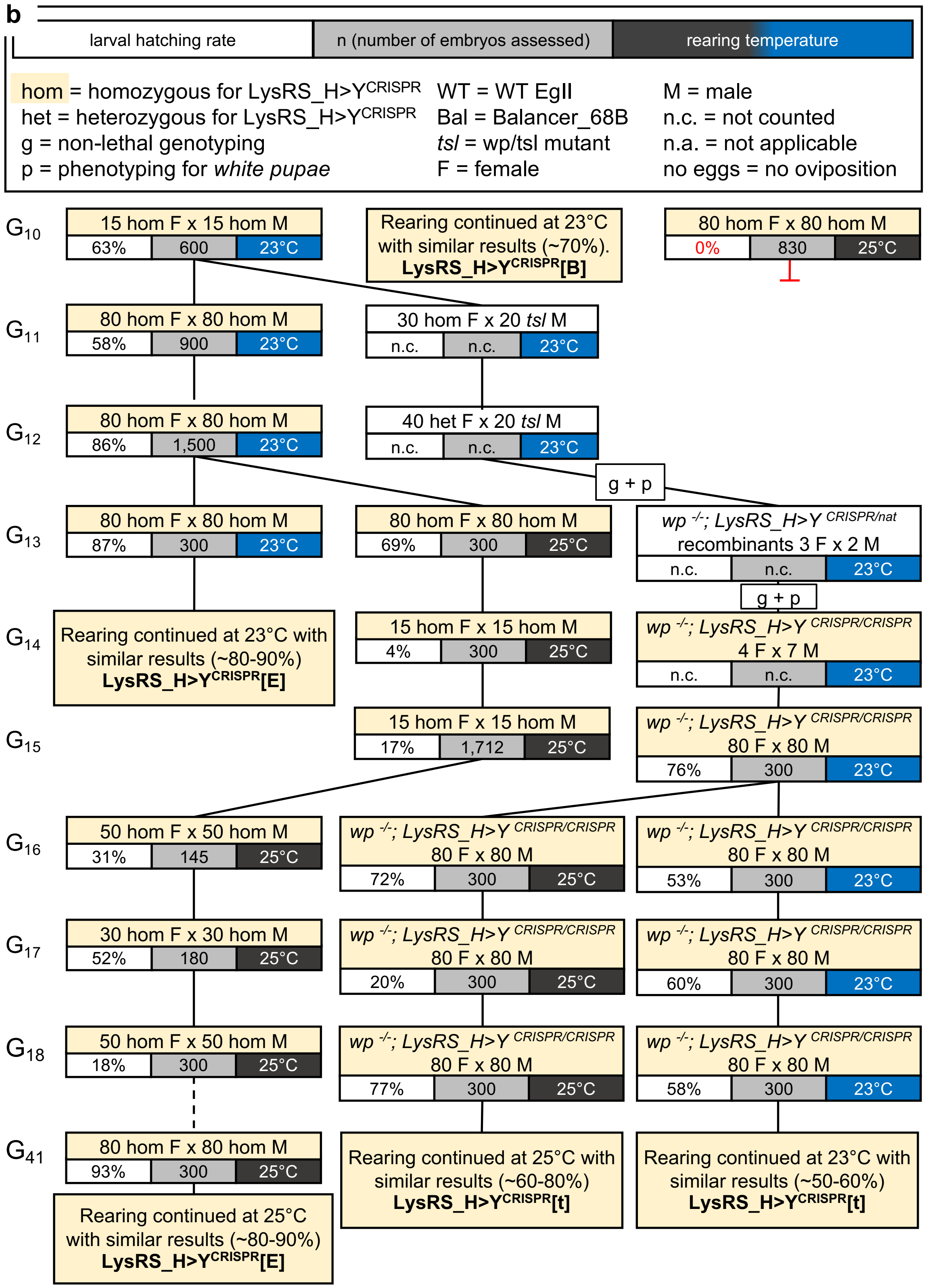

Fig. S6. | Generation and maintenance of homozygous *LysRS_H>Y^CRISPR* mutants. b, The cross setup for generations G_{10}-G_{18} and G_{41}, including the number of flies, genotypes, the average hatching rate, the number of embryos assessed to calculate it, and the rearing temperature.

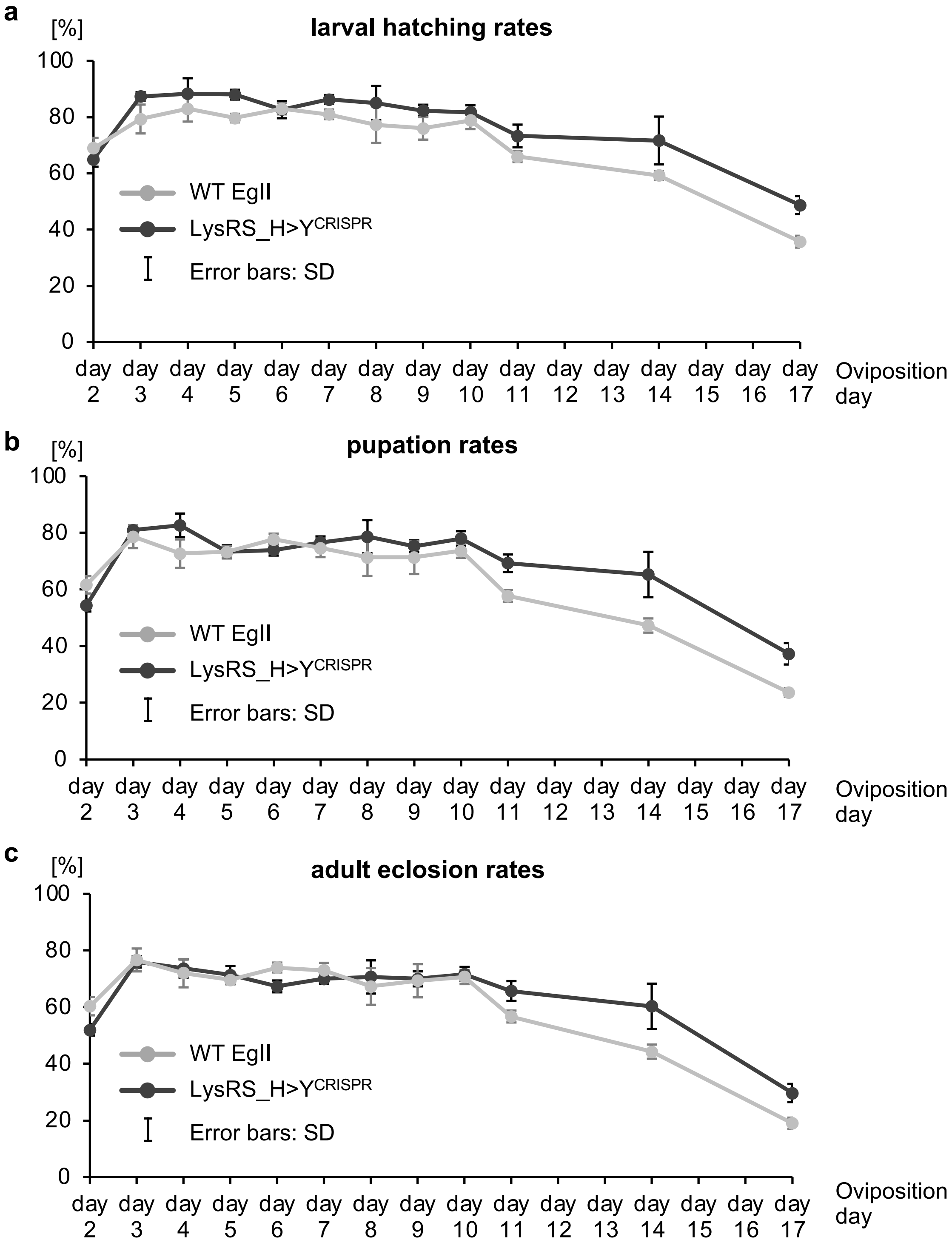

Fig. S7. | Fertility and survival rates of *LysRS_H>Y^{CRISPR}[E]* and WT EgII at 23°C. a, Larval hatching rates. **b,** Pupation rate. **c,** Adult eclosion rates. All data represent egg collections from *LysRS_H>Y^{CRISPR}[E]* mutants and WT EgII flies on different oviposition days (*n* = 3×100 eggs per day and strain). Larval hatching was calculated by counting and subtracting the number of unhatched eggs from the number of collected embryos. Pupal and adult recovery was calculated by dividing the number of pupae or adults, respectively, by the number of collected embryos. Error bars represent the standard deviation (SD) of the measurements.

a

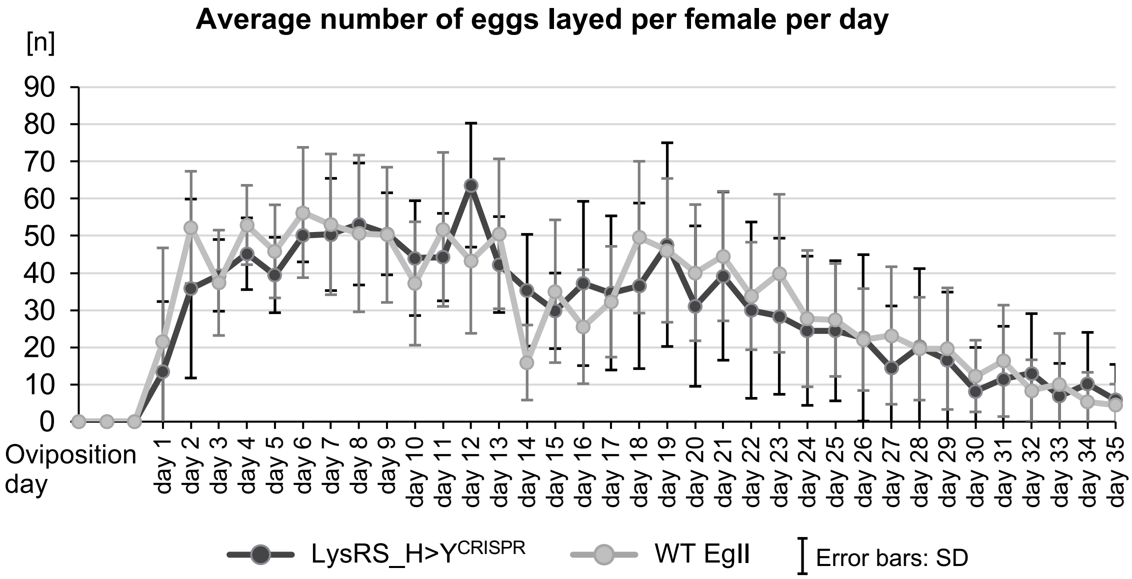

b

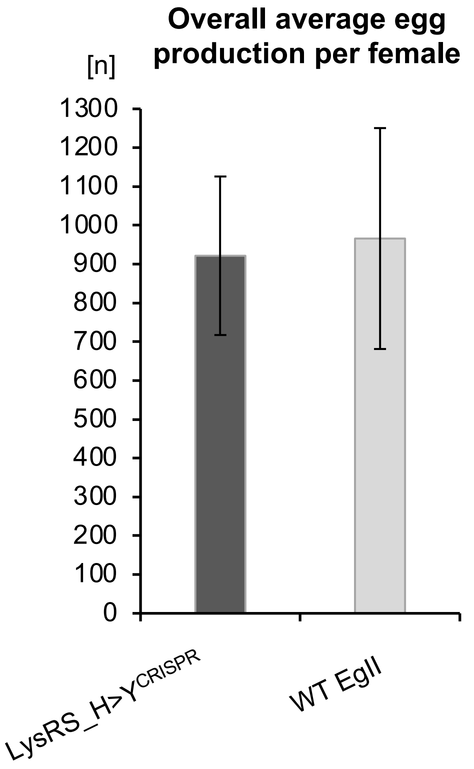

Fig. S8. | Fecundity of *LysRS_H>Y^{CRISPR}[E]* and WT EgII at 23°C. a, The average number (*n*) of eggs laid per female fly per day. The oviposition start (day 1) corresponds to an adult age of 4 days. **b,** The average overall egg production per female (*n* = 11) over all assessed oviposition days (days 1–35), We used 11 cages per strain, single female crosses (one female, three males), and eggs were counted every 24 h. Error bars represent the SD of the measurements.

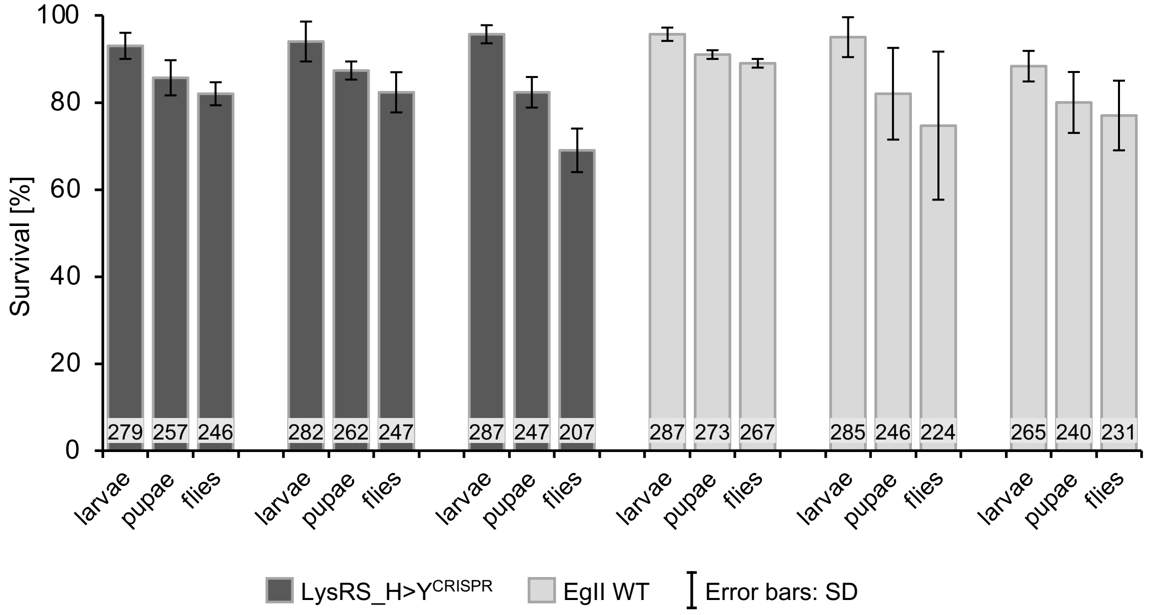

Fig. S9. | Survival rates of *LysRS_H>Y*^{CRISPR}*[E]* and WT EgII at 25°C. Larval hatching, pupation, and adult eclosion rates (shown as a percentage (mean ± standard deviation)) were assessed for *LysRS_H>Y*^{CRISPR}*[E]* mutants and WT EgII strains reared continuously at 25°C for 28 and 495 generations, respectively. Three sets of bars (larvae, pupae, and flies) for each strain are displaying biological replicates. Each individual bar represents three technical replicates (*n* = 3×100 eggs per strain). Larval hatching rates were calculated by counting and subtracting the number of unhatched eggs from the total number of collected embryos. Pupal and adult recovery rates were calculated by dividing the number of pupae or adults by the total number of collected embryos. The absolute numbers of larvae, pupae, and adults are presented within the respective bars. Error bars indicate the standard deviation (SD) of the measurements.

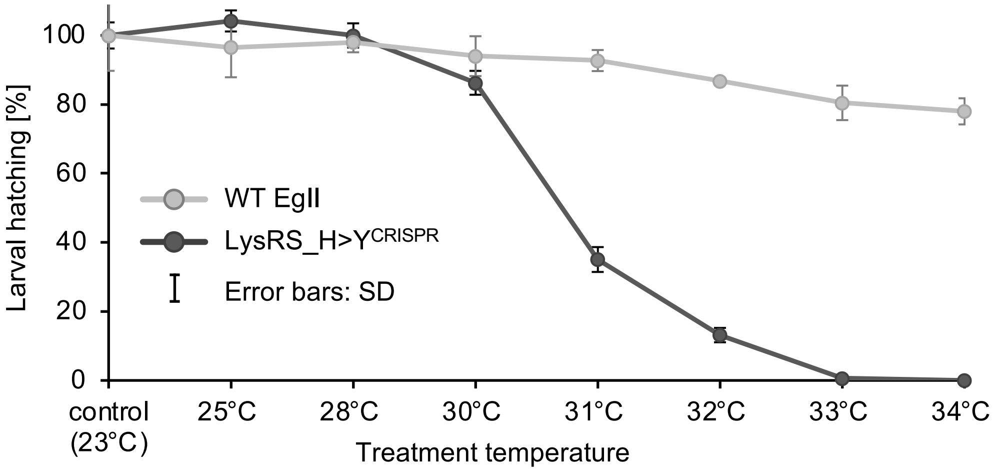

Fig. S10. | Larval hatching rates of *LysRS_H>Y^{CRISPR}[B]* and WT EgII after 24 h treatments at different temperatures, relative to the control at 23°C. *LysRS* mutants show significantly increased lethality at 31°C (one-way ANOVA, $p<0.05$; Error bars represent the SD of the measurements), and full lethality starting at 34°C. The 24-h treatments at 25, 28, 30, 31, 32, 33, and 34°C were applied to embryos of the homozygous *LysRS_H>Y^{CRISPR}[B]* and WT EgII strains, aged 24–29 h (*n* = 3×100 eggs per strain).

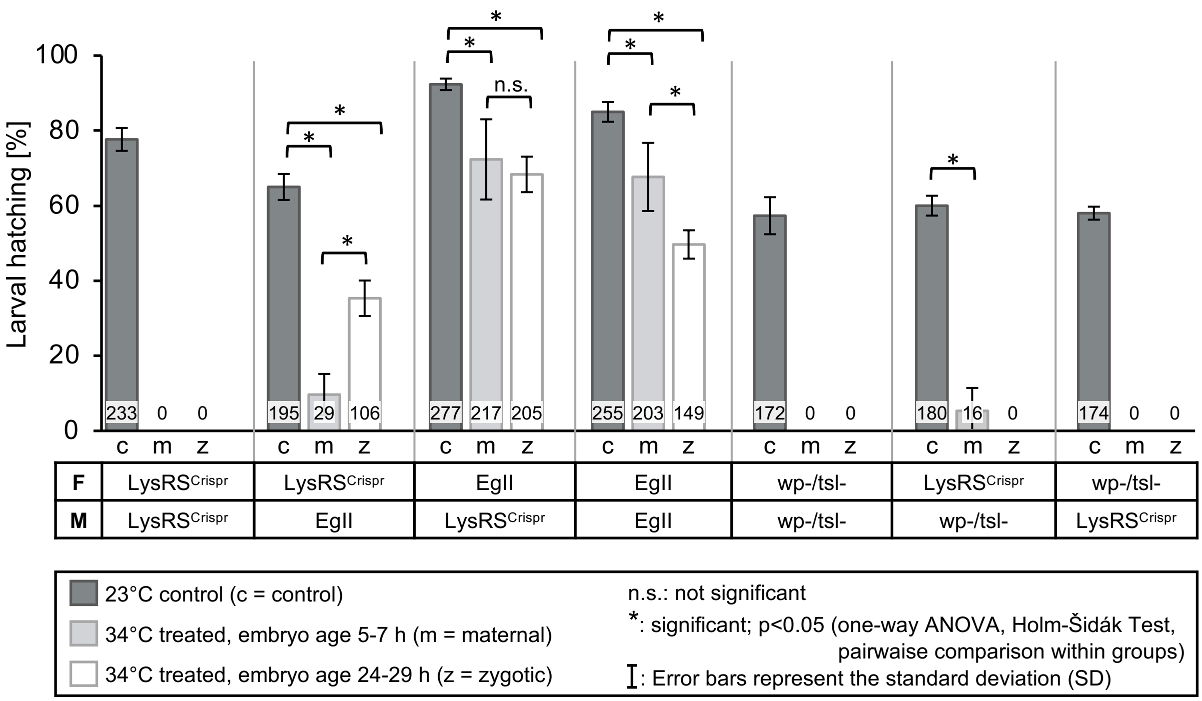

Fig. S11. | Larval hatching rates of reciprocal crosses of *LysRS_H>Y^CRISPR^[B]*, WT EgII and *wp⁻/tsl⁻* mutant flies after heat treatment. The *LysRS* mutants (LysRS^{CRISPR} = *LysRS_H>Y*^{CRISPR}*[B]*) were crossed to WT EgII or *wp⁻/tsl⁻* flies (F = female parent, M = male parent) as depicted below the graph. We collected F_1 embryos (*n* = 3×100 eggs per strain) for 5 h and either kept them at 23°C (c = control), kept them at 23°C for 2 h and then switched to 34°C for 24 h (embryonic age 5–7 h, m = maternal treatment), or kept them at 23°C for 24 h and switched them to 34°C for 24 h (embryonic age 24–29 h, z = zygotic treatment). The larval hatching rate is shown as a percentage (mean ± standard deviation) along with the absolute number of hatched larvae (inside the bar). One-way ANOVA was used to determine whether differences within groups were significant (p<0.05, Holm-Šidák test) or not (n.s. = not significant). Error bars represent the SD of the measurements. The experiment was carried out twice (biological replicates) with similar results.

a

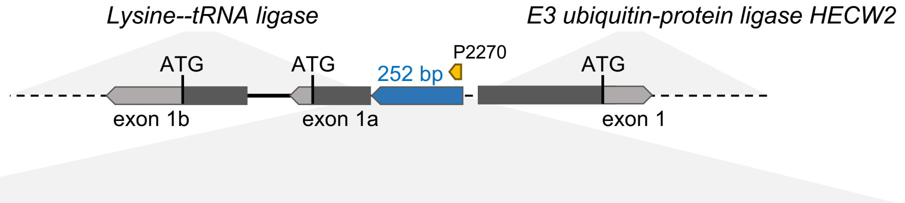

b

*LysRS_5'UTR_*GATAAAATATGGCGTACATACAATATATTGTATGTATGAT
AATTTTTAAATATTTAATAGTTATAGTATGAATGCAAATATATTCTGTATTT
TTCCTTCTTATAACTGATATTGACAAAAAAAACCTTTAATTTTATGTACAT
TTTTCAAAATTATATGTTTGATAGTGTTAAACGCTTTTCTCAAAAATAAAA
GTATTGTGCACCCTTTGTTTTAACAAATGTTTAAATGGTGCTAATTGACA
ACCTAACT…_ *E3 ubiquitin-protein ligase HECW2_5'UTR*

Fig. S12. | The *LysRS* gene and its upstream neighbor *HECW2* encoding E3 ubiquitin-protein ligase. a, Part of the intragenic sequence (252 of 271 bp) between LOC101451725 (*HECW2*) and LOC101451416 (*LysRS*) was used to construct the *mini-LysRS rescue* construct, which was tested for promoter activity (Fig. 4). Untranslated regions (UTRs) are shown in dark gray, coding sequences (CDS) in light gray, and start codons in black. The primer position (P2270) used to amplify the putative promoter region is indicated. Not drawn to scale. **b,** The intragenic sequence used as a putative promoter region in the rescue construct is shown in blue.

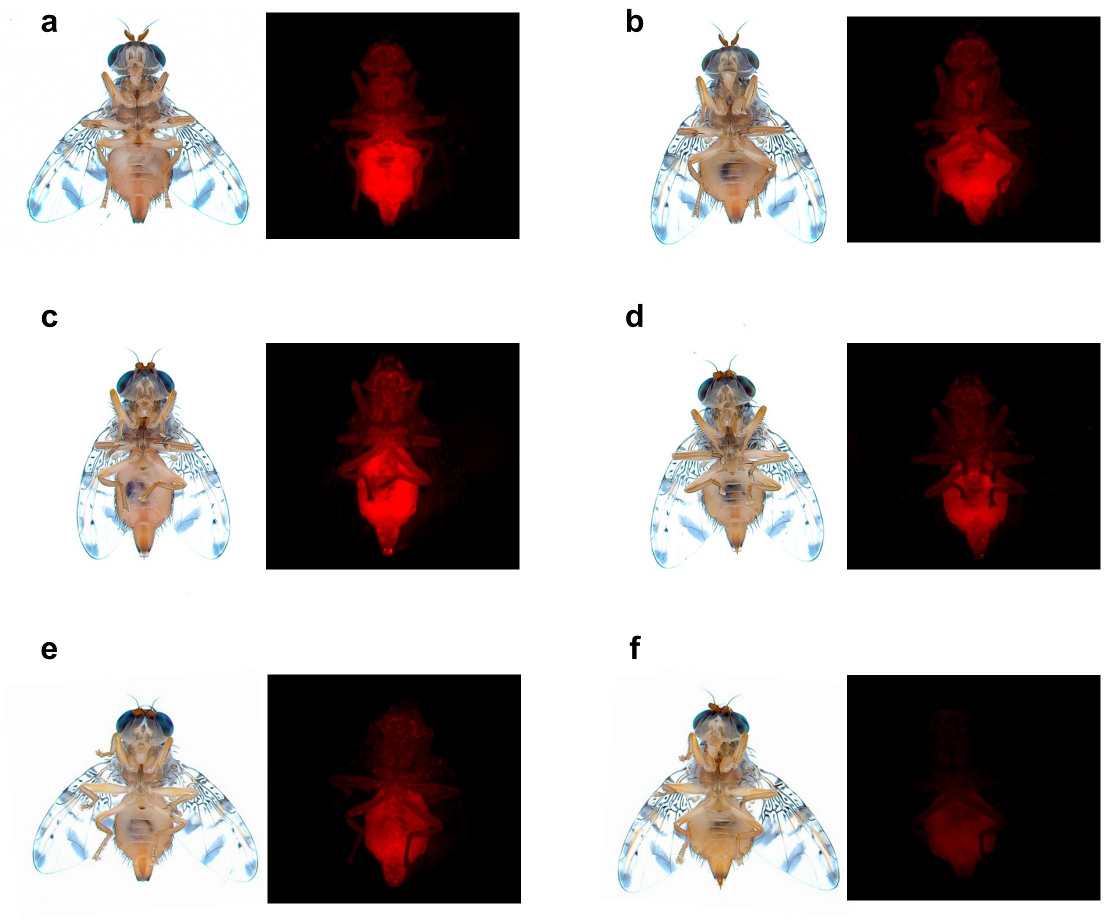

Fig. S13. | Bright-field and fluorescence images of *mini-LysRS rescue* strains. **a,** MG6_m1. **b,** FG3_m1. **c,** FG7_m5. **d,** FG3_m2_m1. **e,** FG3_f1_m1. **f,** M1_m1_m1. All strains are homozygous and have been verified to carry a single autosomal integration event of the *mini-LysRS* rescue cassette.

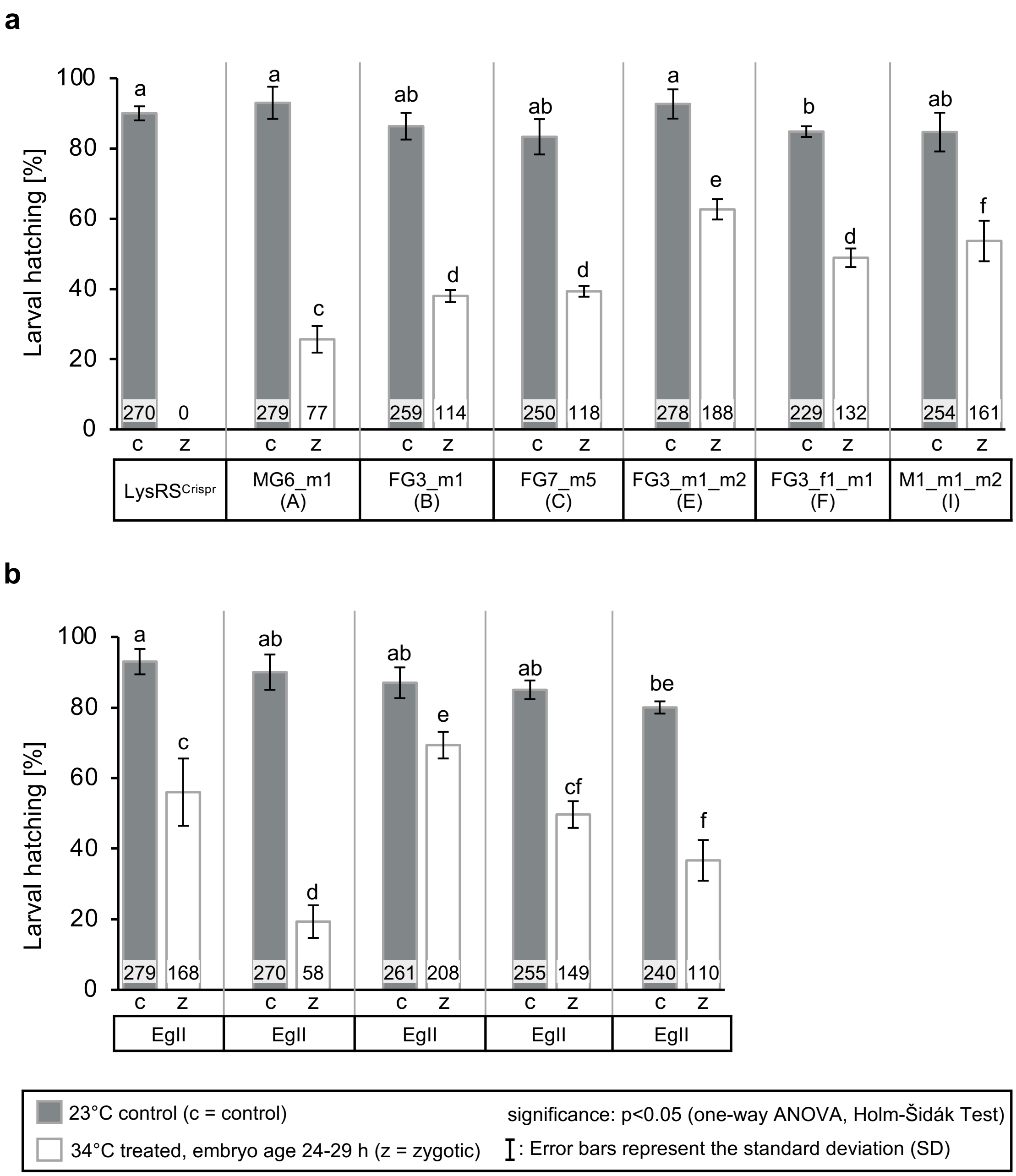

Fig. S14. | Larval hatching rates of *mini-LysRS rescue* and WT strains after heat treatment. We collected 3×100 embryos over a period of 5 h and either kept them at 23°C (c = control) or kept them at 23°C for 24 h and then switched to 34°C for 24 h (embryonic age 24–29 h, z = zygotic treatment). The larval hatching rate is shown as a percentage (mean ± standard deviation) along with the absolute number of hatched larvae (inside the bar). One-way ANOVA was used to determine whether differences within groups were significantly different ($p<0.05$, Holm-Šidák test; indicated by different letters) or not (identical letters). Error bars represent the SD of the measurements. **a,** Homozygous *mini-LysRS* rescue mutant strains with homozygous *LysRS_H>Y^{CRISPR}[E]* as a control. **b**, Homozygous WT EgII strain in five different generations for comparison.

Table S1. | Names and sequences of oligonucleotides used in this study

Name	Sequence (5' – 3')
mfs10	ACGACCGCGTGAGTCAAAATGACG
mfs11	ATCAGTGACACTTACCGCATTGACA
mfs34	CGTACGTCACAATATGATTATCTTTCTAGG
P49	GATCCACAAGGCCCTGAAGC
P50	GCTCCACGATGGTGTAGTCC
P101	AGTAGCTTTGCGTGAAATTCG
P103	CTTCCTGTAAAGCACCAATAGC
P115	GTGTTTTATCGGTCTGTATATCGAGG
P369	AAAAGCACCGACTCGGTGCCACTTTTTCAAGTTGATAACGGACTAGCCTTATTTTAACTTGCTATTTCTAGCTCTAAAAC
P2068	GCAAATATGTCCGAGGCAACC
P2069	CGGTTGCGTTCTGTTAGCTTC
P2070	AAGCCGAACAAAAAGCCAAGG
P2087	TCCTGAATTCACCACGTGCG
P2090	AGACATGTATAAATCCAACCTTTATCTGTGAGCATCCGCAAATAATGTCGCCATTAGCTAAATACTACCGGAGTGAGCCAGGATTAACGGAACGTTTTGAGTTGTTCATCATGAAGAAAGAAGTGTGTAATGCGTATACA
P2091	GAAATTAATACGACTCACTATAGGTTAATCCTGGCTCACTACGAGTTTTAGAGCTAGAAATAGC
P2242	CTAAAATTGCGTCAGTTGTCCG
P2243	GATTACGCCAAGCTTCACATGTTGCCCCATTTGCACTACGGCC
P2244	GATTACGCCAAGCTTCGATTTCCTCTTCGGGTCCTTCGGGCC
P2267	AAACTCATCAATGTATCTTAATTTAAGGGGCAAATAAATATTT
P2268	ACTTATGAGGGTATGGATCAGTTTCTGGGGA
P2269	ATCCATACCCTCATAAGTTTAACGTTAGCA
P2270	ATATATATTTTCTTGTTATAGATATCAAGTTAGGTTGTCAATTAGCACCA
probe *CcHIS*-HEX	HEX-TGCTGATCCGAAAATTGCCA-BHQ1
probe DsRED-FAM	FAM-TCGTTGTGGGAGGTGATGTC -BHQ1

SI References

1. J. Li, A. Handler, Temperature-dependent sex-reversal by a *transformer-2* gene-edited mutation in the spotted wing drosophila, *Drosophila suzukii*. *Sci Rep* **7**, 12363 (2017).
2. K. Eckermann *et al.*, Hyperactive *piggyBac* transposase improves transformation efficiency in diverse insect species. *Insect Biochem Mol Biol* **98**, 16-24 (2018).
3. C. Ward *et al.*, White pupae phenotype of tephritids is caused by parallel mutations of a MFS transporter. *Nat Commun* **12**, 491 (2021).
4. G. Sollazzo *et al.*, Genomic and cytogenetic analysis of the *Ceratitis capitata temperature-sensitive lethal* region. *G3* **13**, jkad074 (2023).
5. G. Sollazzo *et al.*, *Deep orange* gene editing triggers temperature-sensitive lethal phenotypes in *Ceratitis capitata*. *BMC Biotechnol* **24**, 7 (2024).
6. A. Papanicolaou *et al.*, The whole genome sequence of the Mediterranean fruit fly, *Ceratitis capitata* (Wiedemann), reveals insights into the biology and adaptive evolution of a highly invasive pest species. *Genome Biol* **17**, 192 (2016).
7. G. Franz, K. Bourtzis, C. Caceres, "Practical and operational genetic sexing systems based on classical genetic approaches in fruit flies, an example for other species amenable to large-scale rearing for the sterile insect technique" in Sterile Insect Technique: Principles and Practice in Area-Wide Integrated Pest Management (2nd ed.), H. J. Dyck VA., Robinson AS., Ed. (IAEA. CRC Press, Boca Raton, Florida, USA, 2021), chap. 4.3, pp. 575-604.
8. P. Kerremans, G. Franz, Cytogenetic analysis of chromosome 5 from the Mediterranean fruit fly, *Ceratitis capitata*. *Chromosoma* **103**, 142-146 (1994).
9. N. Niyazi *et al.*, Genetics and mating competitiveness of *Ceratitis capitata* (Diptera: Tephritidae) strains carrying the marker *Sergeant*, *Sr*2. *Ann Entomol* **98**, 119-125 (2005).
10. M. Porras, J. Meza, E. Rajotte, K. Bourtzis, C. Caceres, Improving the phenotypic properties of the *Ceratitis capitata* (Diptera: Tephritidae) temperature-sensitive lethal genetic sexing strain in support of sterile insect technique applications. *J Econ Entomol* **113(6)**, 2688-2694 (2020).
11. P. Gourzi *et al.*, The construction of the first balancer chromosome for the Mediterranean fruit fly, *Ceratitis capitata*. *Mol Gen Genet* **264**, 127-136 (2000).
12. M. Scott, D. Kriticou, A. Robinson, Isolation of cDNAs encoding *6-phosphogluconate dehydrogenase* and *glucose-6-phosphate dehydrogenase* from the Mediterranean fruit fly *Ceratitis capitata*: correlating genetic and physical maps of chromosome 5. *Insect Mol Biol* **1**, 213-222 (1993).
